# Supplementary material for: Multiplex base editing to convert TAG into TAA codons in the human genome
Source: Nat Commun. 2022 Aug 2;13:4482. doi: 10.1038/s41467-022-31927-8 (PMC9345975; doi:10.1038/s41467-022-31927-8)
Supplement: Supplementary file 1 — supplementary information [file 41467_2022_31927_MOESM1_ESM.pdf]

Multiplex base editing to convert TAG into TAA codons in the human genome

Yuting Chen[1,2,3]†, Eriona Hysolli[1,2]*†, Anlu Chen[4]†, Stephen Casper[1]†, Songlei Liu[1,2], Kevin Yang[1], Chenli Liu[3]*, George Church[1,2]*

[1]Department of Genetics, Harvard Medical School, Boston, MA 02115, USA.
[2]Wyss Institute for Biologically Inspired Engineering, Boston, MA 02115, USA.
[3]CAS Key Laboratory of Quantitative Engineering Biology, Center for Genome Engineering and Therapy, Shenzhen Institute of Synthetic Biology, Shenzhen Institute of Advanced Technology, Chinese Academy of Sciences, Shenzhen 518055, China
[4]Division of Endocrinology, Diabetes and Metabolism, Beth Israel Deaconess Medical Center, Harvard Medical School, Boston, MA 02215, USA

***Corresponding author. Email: gchurch@genetics.med.harvard.edu (G.M.C); cl.liu@siat.ac.cn (C.L.);eriona.hysolli@gmail.com (E.H.).**

† These authors contributed equally to this work

Supplementary Information

Supplementary Figure 1. Virus resistance schematic.
Supplementary Figure 2. Inducible cytosine base editor (CBE) single clone screening.
Supplementary Figure 3. Determination of stability of gBlocks in plasmid amplification and mammalian cell.
Supplementary Figure 4. Delivery of different number of gBlocks pools into HEK293T CBE stable cell line.
Supplementary Figure 5. Synthesis and golden gate assembly strategy for 43 sgRNAs all-in-one plasmid.
Supplementary Figure 6. Schematic diagram of method_1, method_2 and method_3 for converting TAG to TAA.
Supplementary Figure 7. Basic quality metrics of single-cell RNA-seq of 3 different delivery methods.
Supplementary Figure 8. For each gene target, distribution analysis of modified cells with different editing efficiency.
Supplementary Figure 9. Single clone screening by Sanger sequencing.

Supplementary Figure 10. Chromosome distribution of exonic SNVs in essential genes.
Supplementary Figure 11. Clustering analysis of single cells from three different delivery methods.
Supplementary Figure 12. Analysis of on-target editing efficiency and single cell clusters.
Supplementary Figure 13. Gene expression analysis in highly modified HEK293T clones and lowly modified clones by bulk RNAseq.
Supplementary Figure 14. Karyotype analysis of the highly and lowly modified HEK293T clones.
Supplementary Figure 15. Potential way to optimize framework for converting TAA to TAG.
Table S1. 50 sgRNAs sequences targeting 52 gene sites.
Table S2. Editing efficiency across mapping region when co-transfection of 10, 20, 30 gBlocks into HEK293T evoAPOBEC1-BE4max-NG stable cell line separately.
Table S3. The editing efficiency of C·G to T·A in two negative control (NC) clones, two lowly modified clones (5, 16), and three highly modified clones (19, 21 and 11).
Table S4. Summary of the karyotype analysis of HEK293T highly modified clones (19,21) and lowly modified clones (11,16).
Table S5. Sanger sequence primers used in this study.
Supplementary Sequence. Full sequence of gBlock-PC plasmid.
Supplementary Note. FACS gating examples for mCherry, EGFP and DsRed cell sorting conditions.

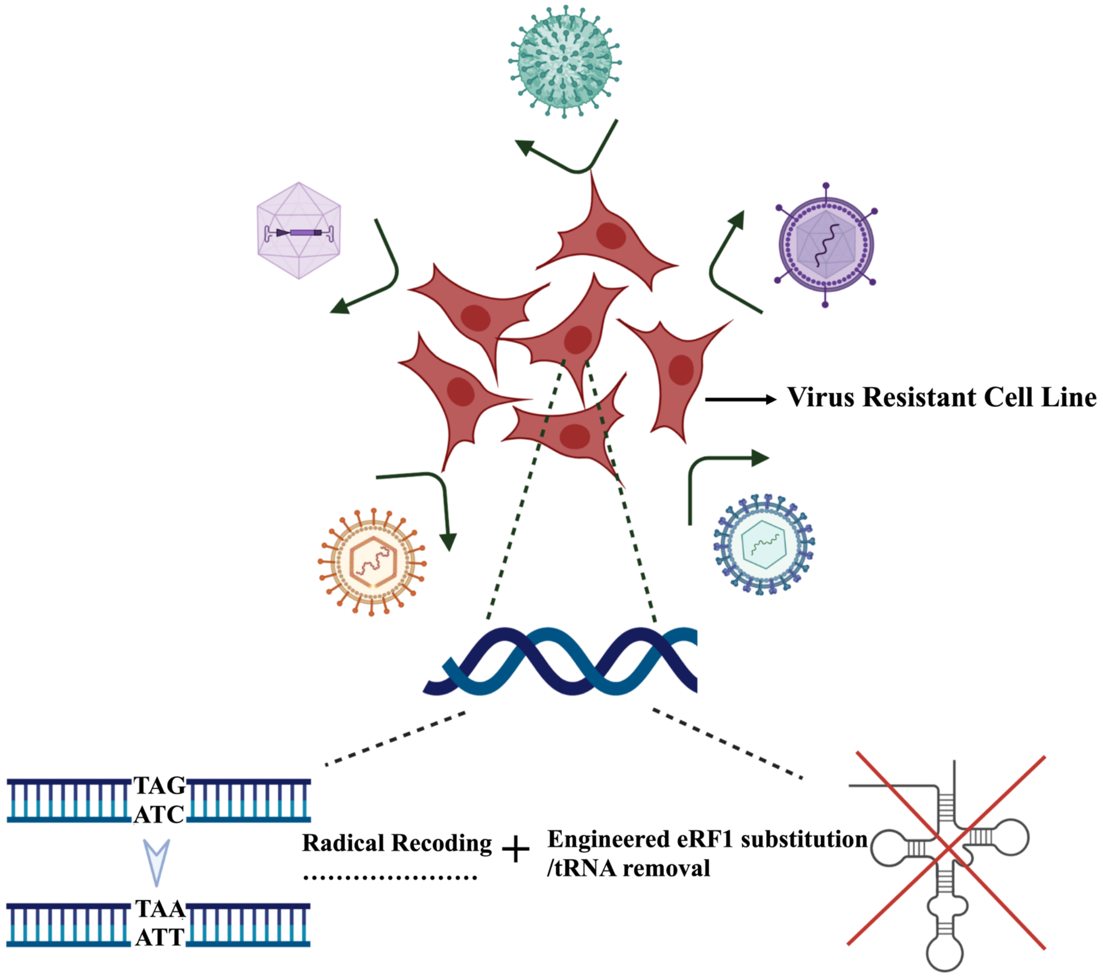

Supplementary Figure 1. Virus resistance schematic. Removal of redundant codons and their respective eukaryotic release factor (eRF) and/or tRNA genes in the genome.

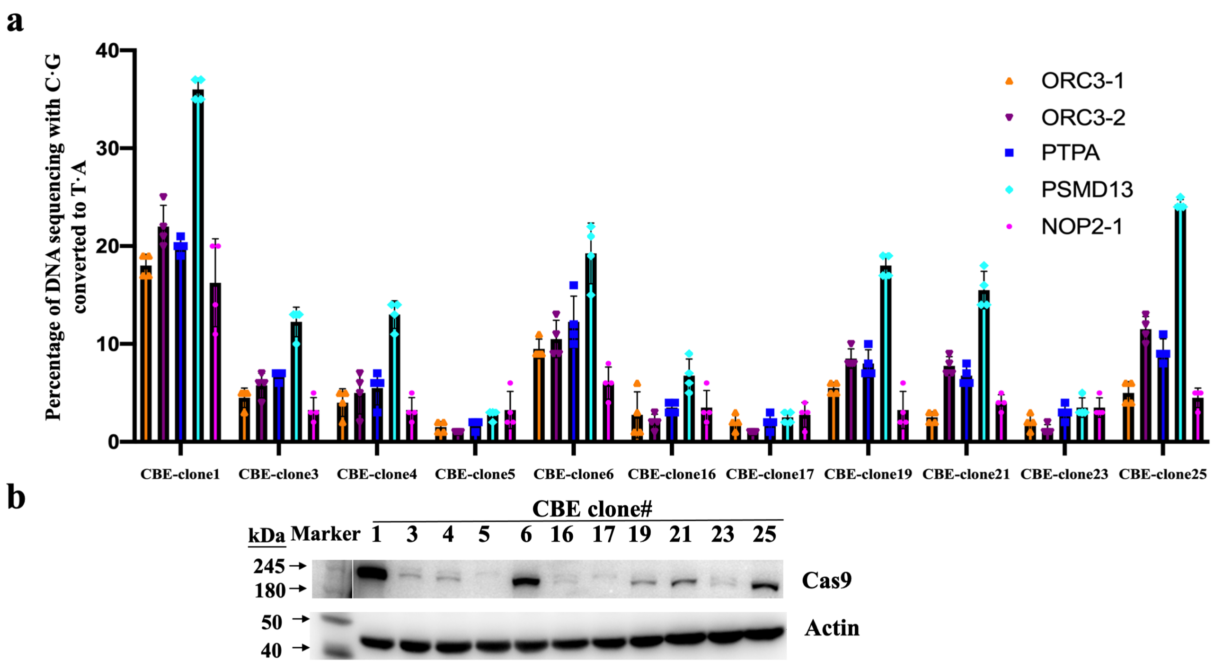

Supplementary Figure 2. Inducible cytosine base editor (CBE) single clone screening. (a) 11 single clones from the drug resistant CBE stable cell population and transfected them with gBlock-YC1. The editing efficiency of all five sites in clone 1 was higher than that of other clones. Values and error bars reflect the means and s.d. of four independent experiments. (b)The protein levels of Cytosine base editor in each clone 5 days after Doxycycline inducible. Anti-Cas9(top) and anti-Actin(bottom) were used. Western blotting images are representative of three independent experiments.

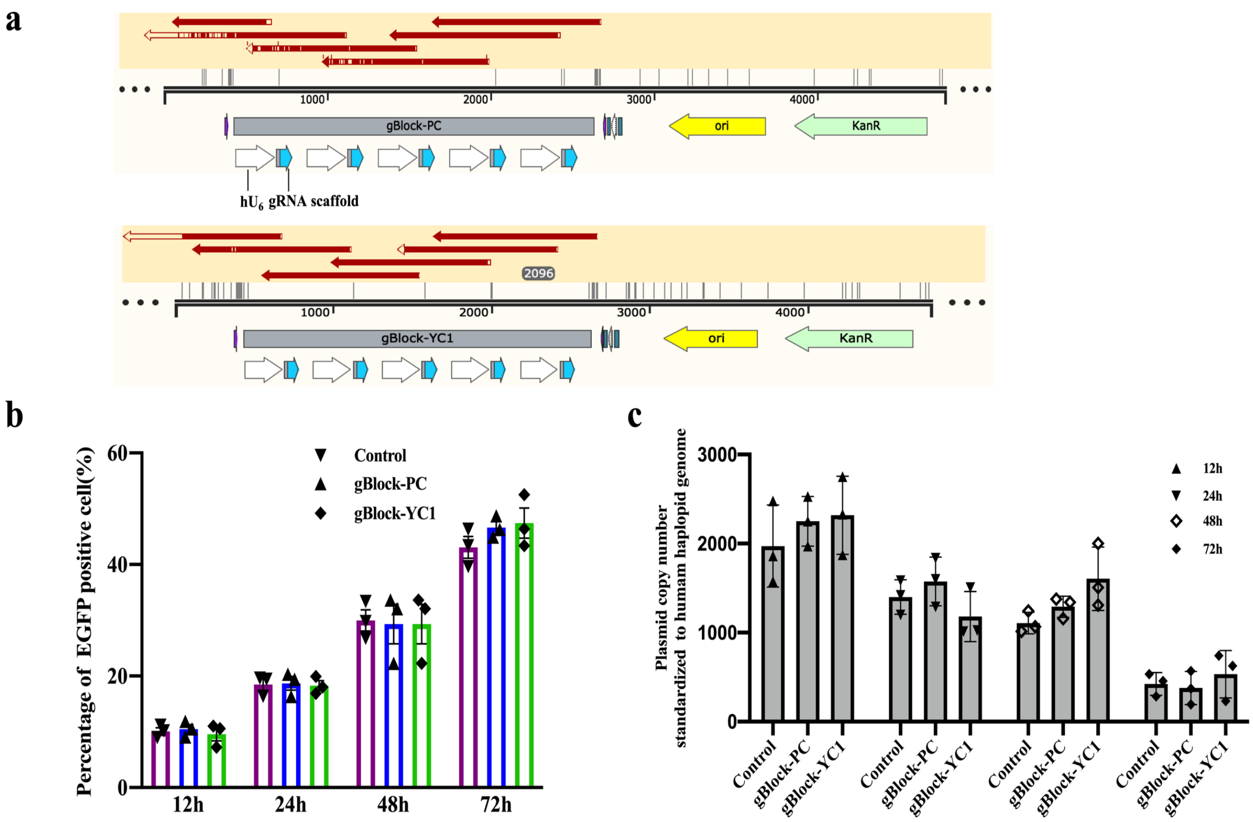

Supplementary Figure 3. Determination of stability of gBlocks in plasmid amplification and mammalian cell. (a) Sequence alignment of gBlock-PC and gBlock-YC1after dozens of transformation rounds. Percentage of EGFP positive cells(b) and copy number(c) of gBlocks and control plasmid when 1ug gBlock-PC, gBlock-YC1and control plasmid (gBlock backbone plasmid containing non-repeating sequence insertions of the same size with gBlocks) with 20ng EGFP were co-transfected into HEK293T at 12, 24, 48, and 72 hours after transfection. Data are mean ± s.d. (n = 3 independent experiments).

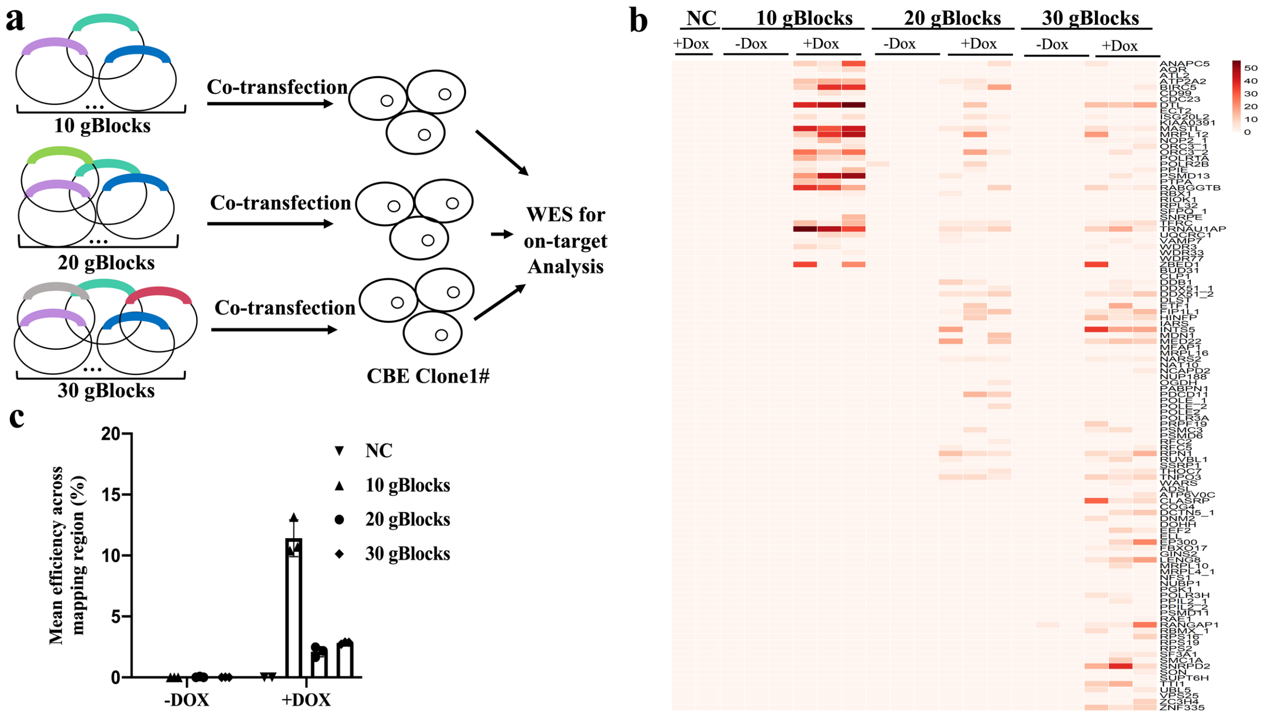

Supplementary Figure 4. Delivery of different number of gBlocks pools into HEK293T CBE stable cell line. (a) Co-transfection of 10, 20, 30 gBlocks into HEK293T evoAPOBEC1-BE4max-NG stable cell line (Clone 1）by lipofectamine 3000 separately. (b) Heatmap of mutation frequency (%) of target "C" in HEK293T cells based on whole exon sequencing under different gBlocks pools and mediums with or without Doxycycline. (c)Mean efficiency across mapping region. Data are mean ± s.d. (n = 3 independent experiments).

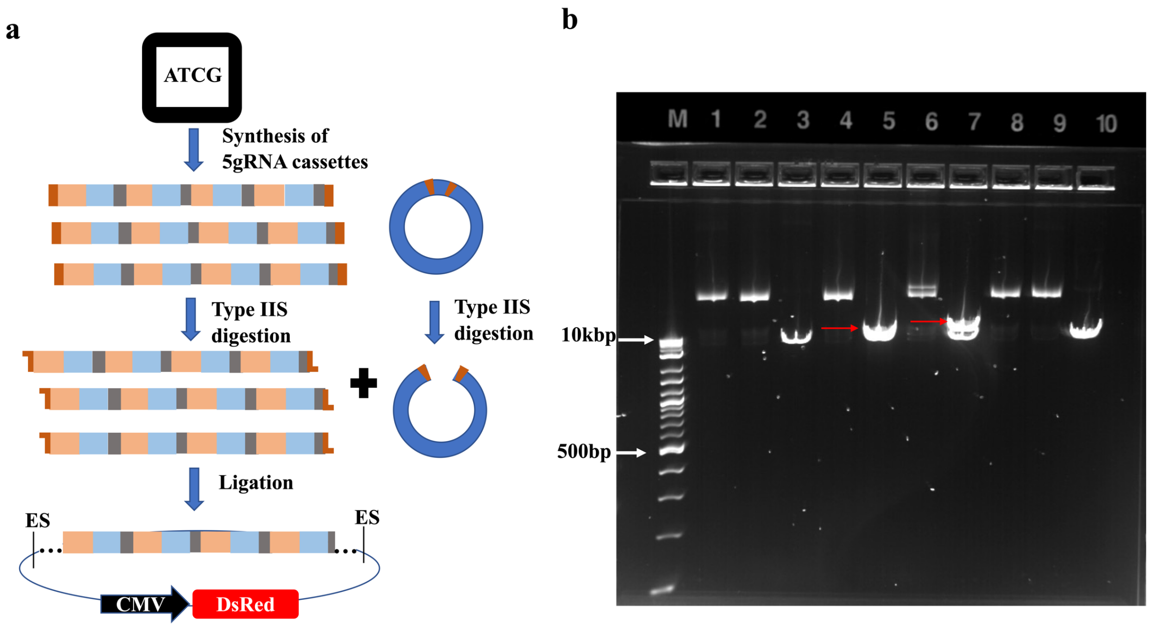

Supplementary Figure 5. Synthesis and golden gate assembly strategy for 43 sgRNAs all-in-one plasmid. (a) workflow of gBlocks assembly. sgRNAs design by software; multiple gRNA array units are synthesized in tandem, and the ends of each synthesized piece of DNA contain overhangs for specified Type IIs restriction sites for Golden Gate; destination plasmid containing Bbs1 site and two spe1 restrictive endonuclease site on both sides of the Bbs1 site, ES, endonuclease site. (b) Agarose gel electrophoresis analysis of the final all-in-one plasmid. DNA ladder was shown on the left. All the plasmids were linearized by endonuclease enzyme spe1. The empty vector on the far right was also shown as a control. Two out of the nine tested plasmids have the right insertion size. Red arrow is 22Kb. Two independent experiments were performed.

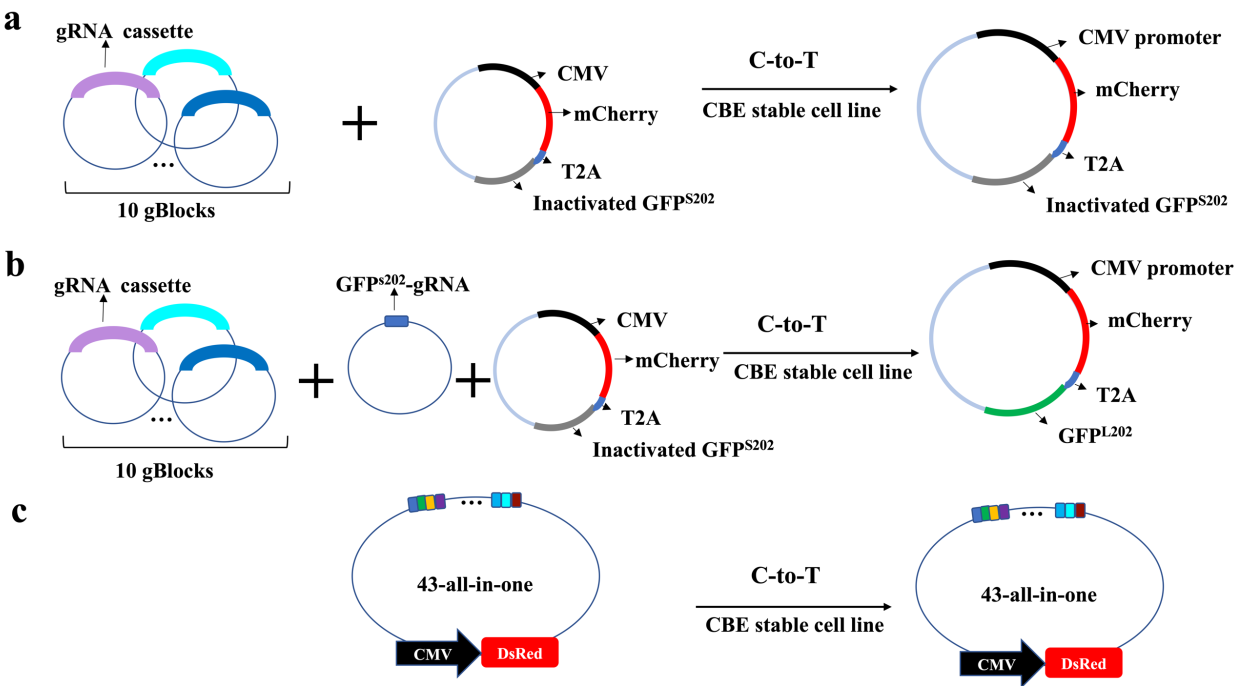

Supplementary Figure 6. Schematic diagram of method_1, method_2 and method_3 for converting TAG to TAA. (a)Method_1: 10 gBlocks + mCherry-inactivated eGFP reporter (transient reporters of editing enrichment), as a transfection reporter; (b) Method_2: 10 gBlocks + mCherry-inactivated eGFP reporter and eGFP cognated sgRNA plasmid, which worked with CBE can turn inactivated eGFP reporter to activated eGFP. (c)Method_3, 43-all-in-one construct contained 43 gRNA array and a CMV promoter driving a Dsred fluorescent reporter as a transfection reporter.

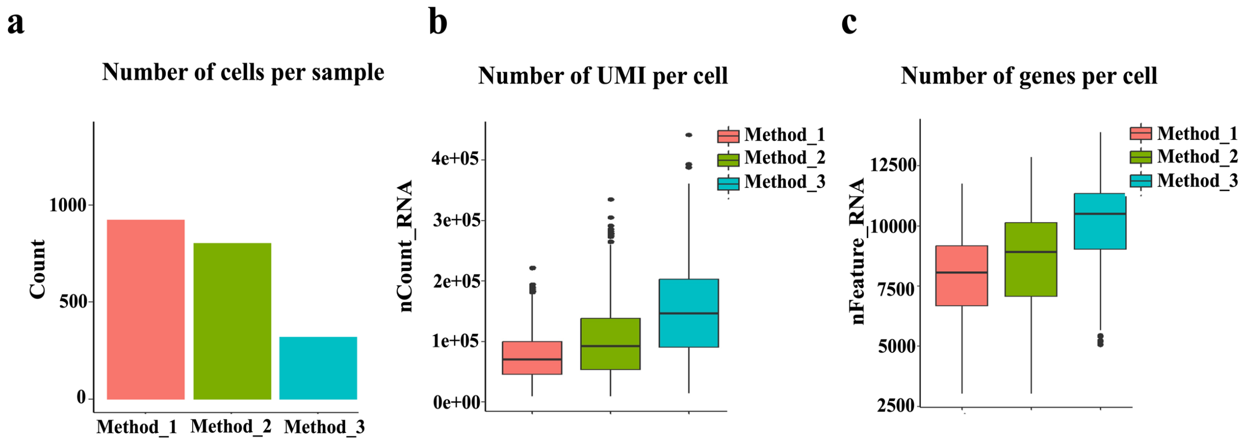

Supplementary Figure 7. Basic quality metrics of single-cell RNA-seq of 3 different delivery methods. (a) Number of cells that were captured. (b) Boxplot for number of UMI per cell. Method 1: minima 9462, maxima 221944, median (line inside box) 70757, first quartile (lower bounds of box) 45901, third quantile (upper bounds of box) 99404. Method 2: minima 9060, maxima 334910, median 92336, first quartile 53482, third quantile 138132. Method 3: minima 13954, maxima 441568, median 147069, first quartile 90692, third quantile 202752. (c) Boxplot for number of genes per cell. Method 1: minima 3022, maxima 11750, median 8063, first quartile 6671, third quantile 9164. Method 2: minima 3023, maxima 12855, median 8920, first quartile 7073, third quantile 10126. Method 3: minima 5056, maxima 13895, median 10508, first quartile 9038, third quantile 11338. In b, c, data represent the mean from two or three independent experiments.

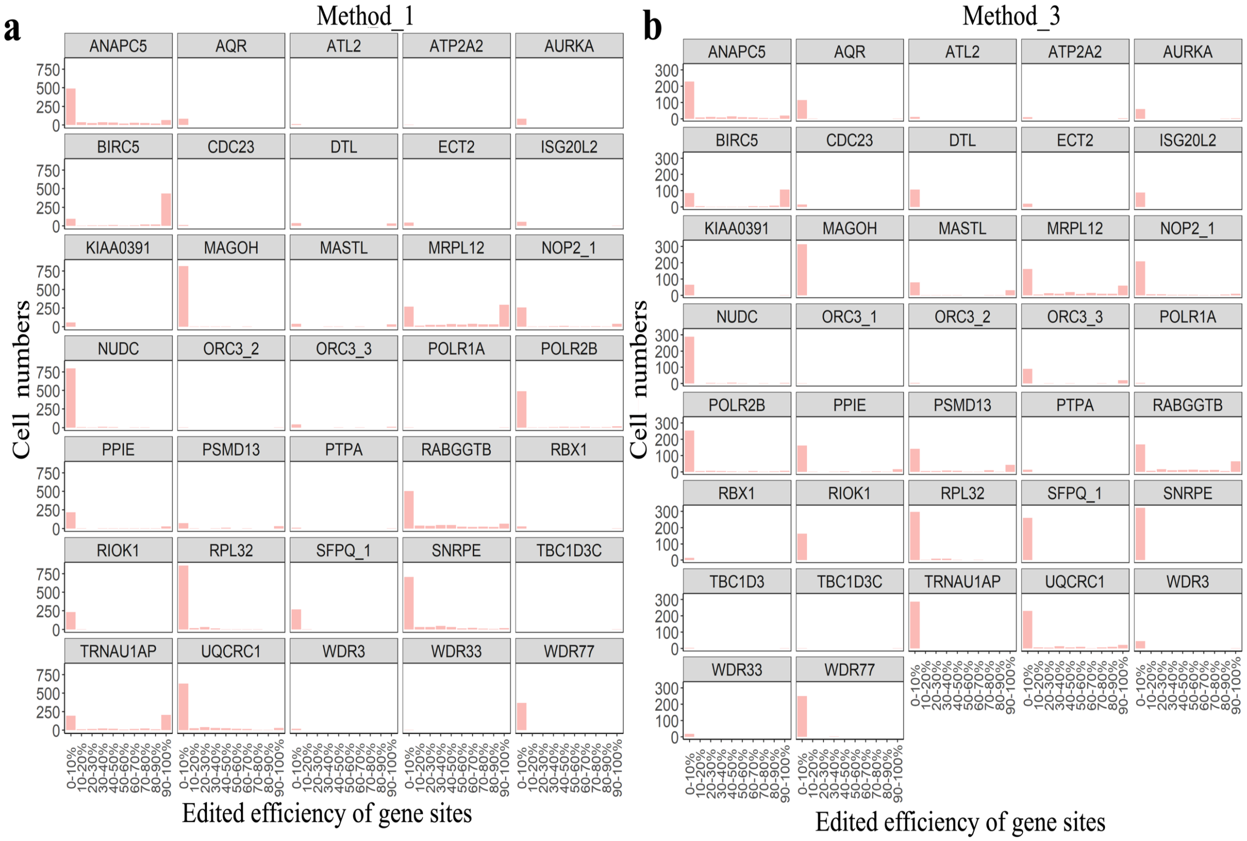

Supplementary Figure 8. For each gene target, distribution analysis of modified cells with different editing efficiency. Counts from method_1 (a) and method_3 (b) were showed in the plot.

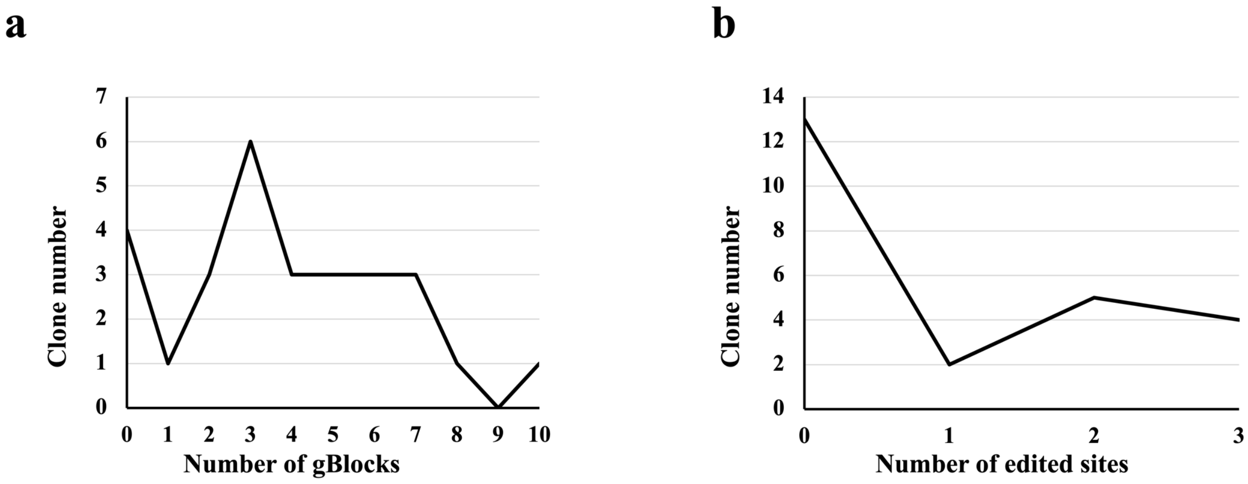

Supplementary Figure 9. Single clone screening by Sanger sequencing. (a) Picked 10 well edited loci (one from each gBlock to validate their delivery) the peak number of gBlocks is 3, and only one clone have all 10 gBlocks. (b) 3 well edited loci for screening and half of clones without any editing and 4 clones have all 3 editing sites.

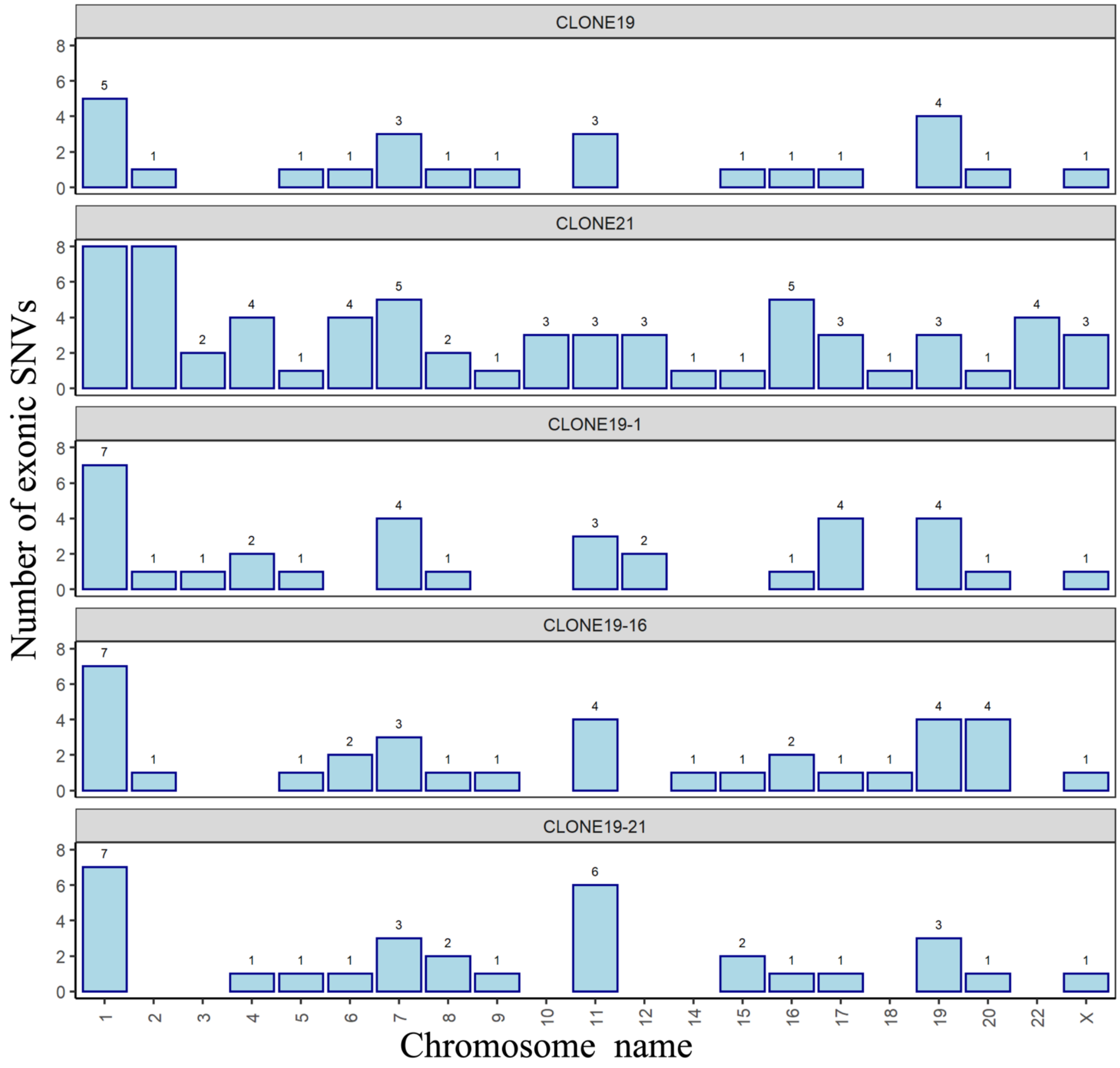

Supplementary Figure 10. Chromosome distribution of exonic SNVs in essential genes. Without the ones in selected 50 essential gene targets. X axis indicates each chromosome, and y axis indicates the count on that chromosome. Number of exonic SNVs in essential genes on each chromosome was marked on top of each bar for better demonstration.

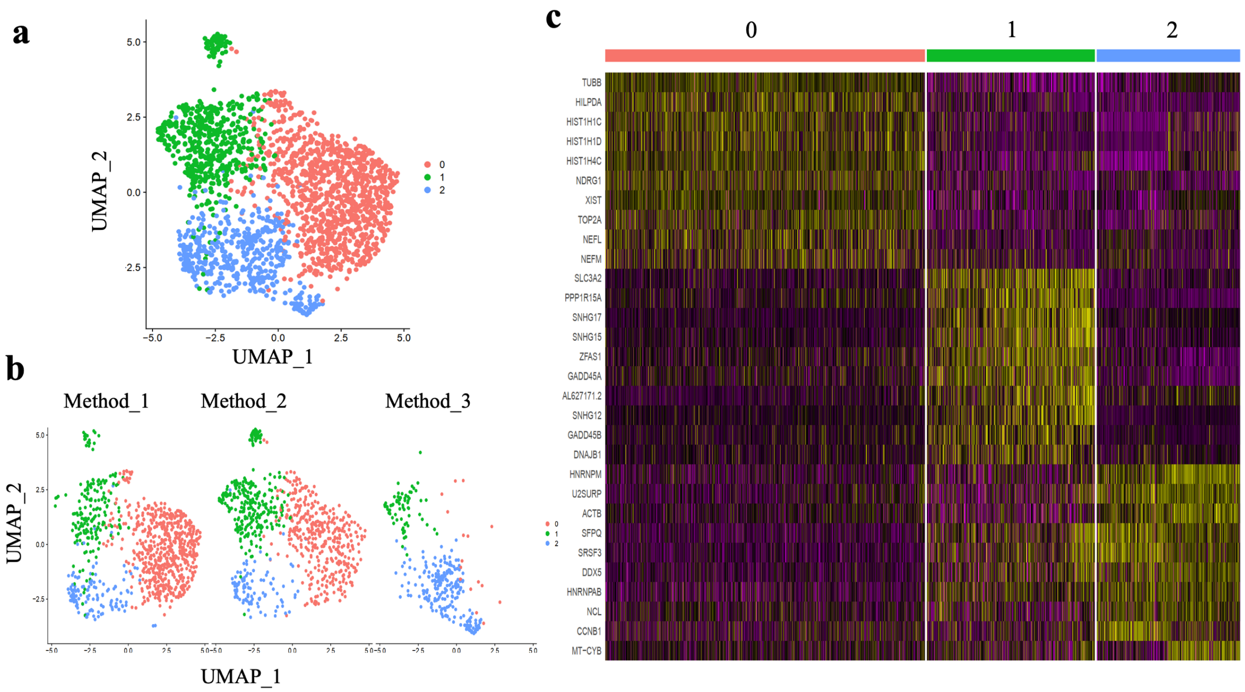

Supplementary Figure 11. Clustering analysis of single cells from three different delivery methods. (a) UMAP of all single cells from three samples, clustering with 0.3 resolution showed three clusters. (b) Distribution of single cells from three samples in the three clusters. (c)Top 10 enriched genes in each of the three clusters.

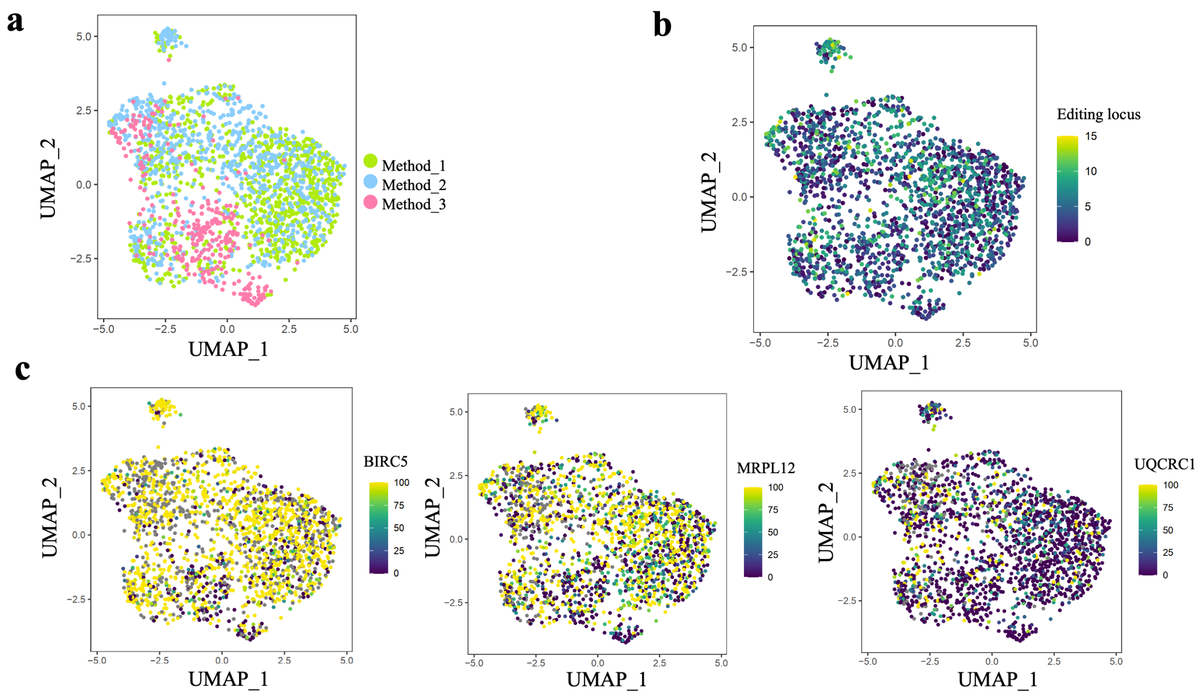

Supplementary Figure 12. Analysis of on-target editing efficiency and single cell clusters. (a) Single-cell UMAP colored by three different delivery methods. (b) Distribution of number of edited loci in single cells on UMAP. (c) Distribution of editing efficiency for representative loci (BIRC5, MRPL12, UQCRC1) on UMAP.

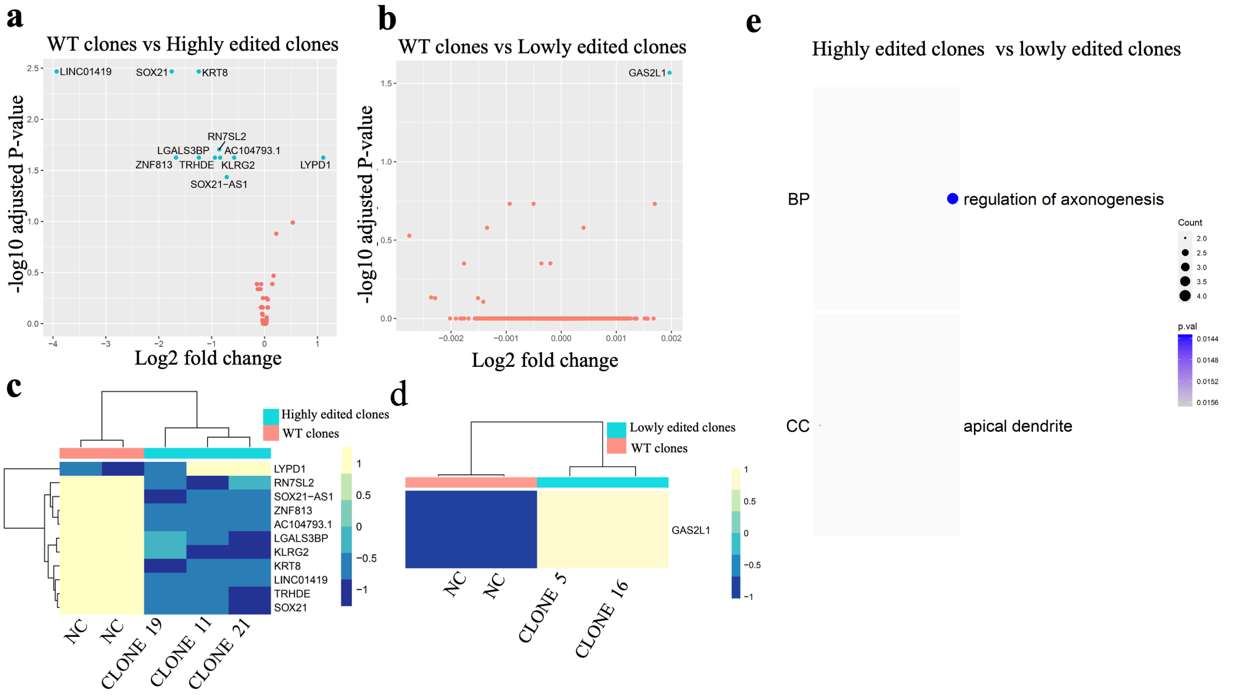

Supplementary Figure 13. Gene expression analysis in highly modified HEK293T clones and lowly modified clones by bulk RNAseq. Volcano plot for differentially expressed genes between wild-type negative control clones and highly modified clones (a) and wild-type negative control clones and lowly modified clones. Heatmap for differentially expressed genes between wild-type negative control clones and highly modified clones(c), wild-type negative control clones and lowly modified clones (d). (e)The GO enrichment analysis of differentially expressed genes (DEGs) between the highly modified clones and lowly modified clones. The enrichment significance is indicated by the color and size of dots as shown on the right.

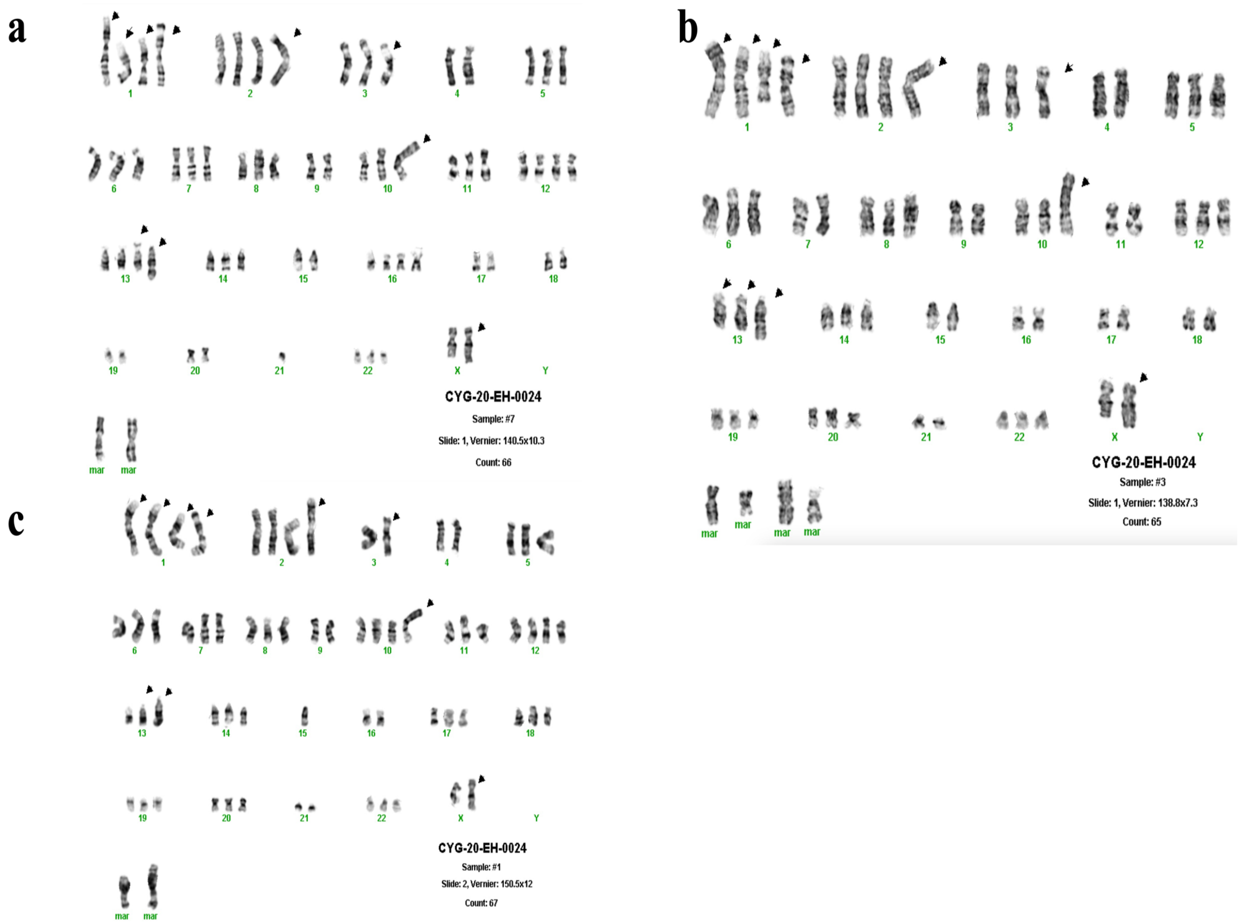

Supplementary Figure 14. Karyotype analysis of the highly and lowly modified HEK293T clones. The chromosomal arrangement of one of highly modified HEK293T clone(a) and one lowly modified HEK293T clone(b) and Wildtype HEK 293T(c) were determined using Karyotype analysis. The black arrows indicated clonal abnormalities.

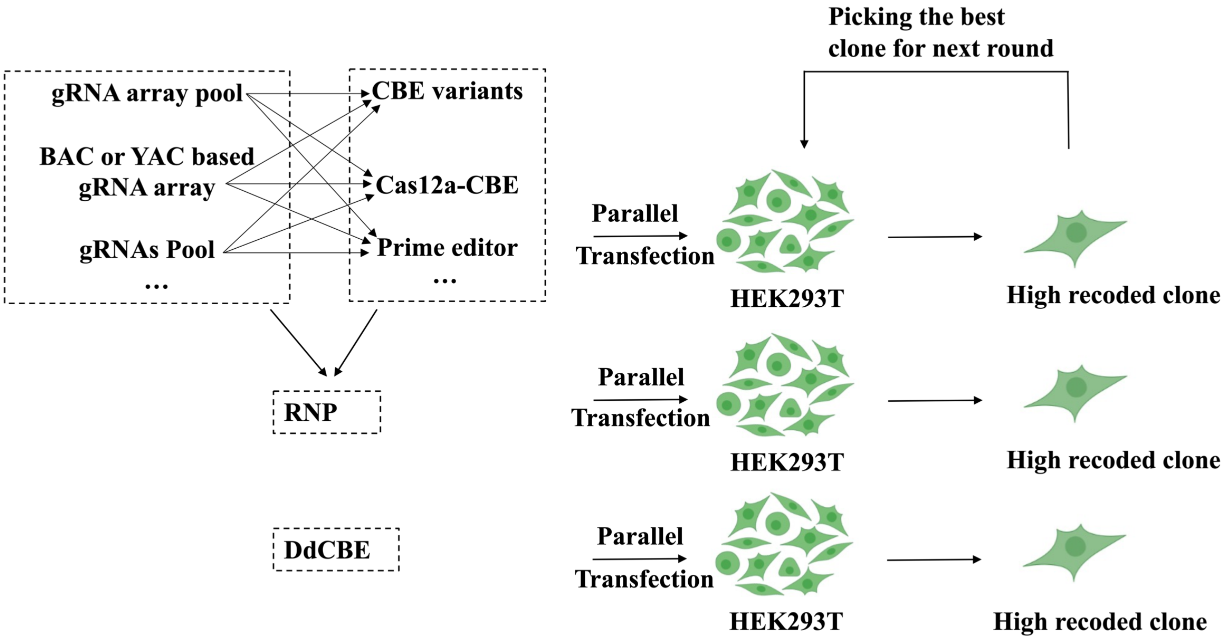

Supplementary Figure 15. Potential way to optimize framework for converting TAA to TAG. BAC, Bacterial artificial chromosome; YAC, Yeast artificial chromosome； RNP, ribonucleoprotein; CBE Variants mean PAMless, less off-target and high efficient Cytosine base editor, Cas12a-CBE,Cas12a-cytidine deaminase fusion； DdCBE, DddA-derived cytosine base editor.

Table S1. 50 sgRNAs sequences targeting 52 gene sites.

Gene	Chr	Strand	Target c/g index	NG-BE gRNA (w/pam)
ORC3	6	f	87651176	CCAAACCTAGCCTATTATCCAG
ORC3	6	f	87667031	AGCTCTAATAAACCGAGCACTG
PTPA	9	f	129147463	CCCTCCTAGCCCGACGTGACAG
PSMD13	11	f	252599	GGCCCTAGGTGAGGATGTCATG
NOP2	12	r	6556992	CCATCTAAGATAGCAGCAGCTG
NOP2	12	r	6558027	CCTAGCTACTTGGGAGTCTGAG
ANAPC5	12	r	121308479	TCTCTAGAGATGGTTTATCAAG
KIAA0391	14	f	35273565	AGAATCTCTATGTCTTTTGGTG
AQR	15	r	34856791	TTTGGCTACTTGGTCTCTTCCG
TBC1D3B	17	r	36165994	GATGCTTCTAGAAGCCTGGAGG
TBC1D3F	17	r	36429011	TTCGTCCCTAGCTCTGAAGGGG
TBC1D3C	17	f	38058007	GATGCTTCTAGAAGCCTGGAGG
TBC1D3	17	f	38181973	GATGCTTCTAGAAGCCTGGAGG
BIRC5	17	f	78223656	CCTTTCCTAAGACATTGCTAAG
MRPL12	17	f	81707239	TGGAGGCTACTCCAGAACCACG
NLGN4Y	Y	f	14841261	GAAAAGCTATACTCTAGTGGTG
SRY	Y	r	2786988	TGTCCTACAGCTTTGTCCAGTG
WDR3	1	f	117959446	TTCAGTTCTAAGTCAACGTTAG
ECT2	3	f	172818768	ATCTCCTAATTCTTCACAAATG
RPL32	3	r	12836093	TGCCTACTCATTTTCTTCACTG
TFRC	3	r	196027173	ATGGTGGCTATCCACGATGGAG
POLR2B	4	f	57030987	ATAGCTAAACACTCATCATTCG
CDC23	5	r	138188977	GCCAACTATGGCGTGACAGAAG
RIOK1	6	f	7417440	TCATTCTATTTGCCTTTTTTCG
ORC3	6	f	87667122	GCTTTCTAGCAGCCTCCCCATG
MASTL	10	f	27186535	TTGTGCTACAGACTAAATCCAG
ATP2A2	12	f	110347411	ACAACTAAAGTTCTGAGCTAAG
AURKA	20	r	56370157	GATTCCTAAGACTGTTTGCTAG
RBX1	22	f	40972487	CTTTTCCTAGTGCCCATACCTG
LOC105373102	Y	f	1396391	CAAGGCTAAGTCCCACGTGCAG
CD99	Y	f	2740803	CAATCTTCTATTTCTCTAAAAG
ZBED1	Y	r	2488634	TCCTCGCTACAGGAAGCTGCTG
VAMP7	Y	f	57128470	TCTTTCCTATTTCTTCACACAG
UTY	Y	r	13249785	GAAACAGCTACAAAACCAGTGG
PPIE	1	f	39763741	GAGCTCTACGTCAGCTTCCAGG
NUDC	1	f	26946180	GGGCTAGTTGAATTTAGCCTTG
WDR77	1	r	111441229	CCAATCTACTCAGTAACACTTG
SFPQ	1	r	35184455	CATCTAAAATCGGGGTTTTTTG
SFPQ	1	r	35186946	ACACACCTAAGTTGTGAAAATG
NSL1	1	r	212727141	CTCTCCTAAACTGCCCCTAGAG
RABGGTB	1	f	75794649	TGAATCTAGCTCACTAGCTCAG
ISG20L2	1	r	156723348	ACTGCCACTAGTCTGTAGGGGG
DTL	1	f	212102939	TAGAATCTATAATTCTGTTGAG
MAGOH	1	r	53227044	AGTCTAGATTGGTTTAATCTTG
ZBTB8OS	1	r	32633309	GAAGCTAGGAGTTCAAGACTAG
TRNAU1AP	1	f	28577635	GCCTGGCTACATCATGGCAGGG
SNRPE	1	f	203869931	ATTTCTAGTTGGAGACACTTTG
MTOR	1	r	11185573	GCACTCTAGCCTGAACAGAGTG
POLR1A	2	r	86027422	GTAGCTGCTATCTCAGAGGCTG
ATL2	2	r	38296482	TACTGTCTAATTTTTCTTCTTG
WDR33	2	r	127706492	CTCCGTCTAAGGAGCTGGAACG
UQCRC1	3	r	48599127	TCCCGCCTAGAAGCGCAGCCAG

Table S2. Editing efficiency across mapping region when co-transfection of 10, 20, 30 gBlocks into HEK293T evoAPOBEC1-BE4max-NG stable cell line separately.

Sample name	Group	Mean	SD	Min	Max	SE
S1_9	NC	0	0	0	0	0
S2_13		0	0	0	0	0
S2_7	10 gBlocks-DOX	0	0	0	0	0
S2_8		0	0	0	0	0
S2_9		0	0	0	0	0
S2_10	10 gBlocks+DOX	10.4	14.49	0	55.77	2.45
S2_11		10.7	15.14	0	46.15	2.56
S2_12		13.15	16.49	0	55.32	2.79
S3_1	20 gBlocks-DOX	0.08	0.66	0	5.56	0.08
S3_2		0	0	0	0	0
S3_3		0	0	0	0	0
S3_4	20 gBlocks+DOX	1.66	3.72	0	18.18	0.44
S3_5		2.48	4.57	0	21.05	0.54
S3_6		2.17	3.98	0	18.18	0.47
S3_7	30 gBlocks-DOX	0	0	0	0	0
S3_8		0.03	0.34	0	3.57	0.03
S3_9		0	0	0	0	0
S3_10	30 gBlocks+DOX	2.89	6.38	0	35.29	0.61
S3_11		2.72	5.35	0	37.88	0.51
S3_12		2.88	5.23	0	25.58	0.5

Table S3. The editing efficiency of C·G to T·A in two negative control (NC) clones, two lowly modified clones (5, 16), and three highly modified clones (19, 21 and 11).

	NC	CLONE 19	CLONE 21	CLONE 19-1	CLONE 19-16	CLONE 19-21
ANAPC5	0	97.14	52.73	100	100	100
AQR	0	0	0	0	36.36	0
ATL2	0	0	0	0	0	0
ATP2A2	0	56.25	47.5	38.46	51.72	100
AURKA	0	0	0	0	0	0
BIRC5	0	79.07	100	100	100	100
CDC23	0	0	0	0	0	0
DTL	0	100	0	100	100	100
ECT2	0	0	0	0	0	0
ISG20L2	0	35.48	0	17.07	18.97	52.87
KIAA0391	0	0	0	0	0	0
MAGOH	0	0	0	0	0	0
MASTL	0	100	100	100	100	100
MRPL12	0	97.37	93.1	95.12	86.49	93.62
NOP2_1	0	35	60.71	40	69.7	66.67
NUDC	0	0	0	0	32.26	0
ORC3_1	0	25.93	0	25	35.71	66.67
ORC3_2	0	60.61	100	62.5	100	100
ORC3_3	0	96.15	100	100	100	100
POLR1A	0	0	36.96	34.15	0	34.29
POLR2B	0	33.33	0	41.67	65	100
PPIE	0	84.44	66.67	78.79	100	90.24
PSMD13	0	61.76	100	100	96.67	100
PTPA	0	33.33	69.57	25.81	51.43	31.71
RABGGTB	0	68.18	100	69.88	100	78.48
RBX1	0	46.94	1.69	15.79	51.39	57.5
RIOK1	0	0	0	0	0	0
RPL32	0	0	27.5	0	36	0
SFPQ_1	0	0	0	0	0	0
SNRPE	0	51.16	0	26.47	56	100
TBC1D3	0	100	100	100	100	100
TBC1D3B	0	80.77	100	59.38	75	77.14
TBC1D3C	0	100	100	100	100	100
TFRC	0	57.89	100	47.83	100	100
TRNAU1AP	0	81.82	0	64.77	71.91	72.62
UQCRC1	0	64.29	2.13	100	55.26	100
WDR3	0	62.9	23.4	61.29	82	100
WDR33	0	0	0	0	2.27	0
WDR77	0	1.56	0	0	0	15.07

Table S4. Summary of the karyotype analysis of HEK293T highly modified clones (19,21) and lowly modified clones (11,16).

	HEK293T	CLONE 52	CLONE 53	CLONE 39	CLONE 59	CLONE 69	293T(CYG-18-PK-0040)	293T (CYG-18-PK-0021)
-X		x	x		x			
add(X)(q28)	x	x	x	x	x	x	x	
der(X)add(X)(p11.2)add(X)(q28)								
add(1)(p36.1)	x	x	x	xx	x	x	x	
add(1)(q42)	xx	xx	xx	xx	xx	xx	xx	
del(1)(q31)	x	x	x	xx	x	xx	x	x
-2								x
i(2)(q10)	x	x	x	x	x	x		
-3				x				
add(3)(p24)	x	x	x		x	x	x	
del(3)(p22)								x
add(3)(q12)							x	
-4	xx	x	x	xx	xx	x	x	x
add(4)(p16)		x						
add(7)(q36)					x			
-7			x				x	
-8	x	x	x	x	x		x	
-9	x	x		x		x		
add(9)(q34)		x						x
add(10)(p13)	x		x	x	x	x	x	
-10		x						
add(11)(q23)				x	x			
-11			x	x				
-12				x				
add(13)(p11)	x	x	x	x	x	x	x	x
add(13)(q34)	x	x	x	x	x	x	x	
-13			x					x
-14		x						
-15	x	x	x	x	x	x	x	x
-16				x				
-17			x	x		x		
-18	x	x	x	x	x	x	x	x
-21	x	x	x	x	x	x	x	x
-22		x						
mar1	x	x						
mar	1-4	1-6	3-5	4-5	2-4	1-4	1-4	3-5

Table S5. Sanger sequence primers used in this study.

Gene	Left primer (5'-3')	Right primer (5'-3')
ORC3_1	CCCTGGCAGTTTACATCTGAGT	AGCAGCATGTGAAGGATTAGCT
ORC3_2	ATACAGCACGGCCAGTTTCC	CTGGTCACCTTTTTCTCTTAAGCT
PTPA	CTTCCTCACAGTGCCTGGAG	ACAGCCTCTCATCAAACGGG
PSMD13	CTGCGTGTCTTAACGTCCCT	AGCTTCATTGGCTGCAAACG
NOP2_1	TGAGAAAGCTGCCTTCCAGA	AGGCAAGAGTTCCAACCTGG
NOP2_2	GACGGTGTCTTGCTTTGTCG	GAGGCCAGGAGTTCAAGACC
ANAPC5	ACGTCGTTTACTTCCAGGCC	AGGGAGAGAGGGGACATGAA
KIAA0391	AACAACTGGAGACTCGTGGC	TCTTCAGGACAGAGGCAATGC
AQR	CACTCCAGAAGCCATCCCTG	TGACAGTAAAATGGCAGAAGCA
TBC1D3B	TAGGGACGAACAGCAGTGTG	TGTCTCTCAAGCTGCACTCT
TBC1D3F	CTAGGGACGAACAGCAGTGT	TCTCTCTCGAGCTGCACTCT
TBC1D3C	CTAGGGACGAACAGCAGTGT	TCTCTCTCGAGCTGCACTCT
TBC1D3	CTAGGGACGAACAGCAGTGT	TCTCTCTCGAGCTGCACTCT
BIRC5	CCATGGATTGAGGCCTCTGG	ACAGGCAGAAGCACCTCTG
MRPL12	CAATGTCGCCAAAGCTGAGG	CGCAGGCAATTCTCCCAAAC
NLGN4Y	TTGATGGGGATGCAGCCTTT	TGGCACAAAAACATTCCTGGT
SRY	GGGATGACTGTACGAAAGCCA	AACATAAGAAAGTGAGGGCTGT
WDR3	ATCTCAAGAGGGAATGCGAGG	ACGCCAACTCTTCTGTGCTT
ECT2	TGTACGTCATTCTGCTTTCCGA	TGTTCCTTTTTTTTTCCCGCA
RPL32	TGGCCATCAGAGTCACCAAC	ATGGGAGATTCCAAAGGGGC
TFRC	CCCAGGAAGGTCCACAGATG	AACCCTTTCTCAAGCTTTGCT
POLR2B	TGCCTTACGCATGCAAACTA	ACAGAAGAAAAGCATGCATATCAT
CDC23	CAAGGCGAGACTCCTACCAC	AGGTCCTTGGAACAGACGTG
RIOK1	GGTCAAGGAAGCCCAGAGAG	CACAGACAGTGCCACGATGA
ORC3	GCCTCCAGTGCTCGGTTTAT	TCTCAACAAAACAGGCTCCTCT
MASTL	ACCTCCTATTTGAAGCCAGGA	TCTGTGAAAGCTGTAACGTACA
ATP2A2	TCGTAAGTGGCTTACCTGGG	TACCAGGCCAGCAGAAACTT
AURKA	CAGAGGCCAATGCTCAGAGA	GAGGGCAGCAGTCAATGGTA
RBX1	CGACAGCCAAGCTAGTGTCA	AGGTAACAGCAGGGAAAGTCA
LOC105373102	TGGATGGATGGATGGATGGATG	GCCCACCTGATCCTCCTTTC
CD99	AGGAACTGTGTCCACGTGAG	CAGAAGGCCTCCATCTCTGC
ZBED1	TTCTGTATGAGAACGCCCGG	CAGCAAAGCATCCAATGGGC
VAMP7	AATTGCTCTCCTCGTCCCTC	GAGGGTTGAAGAGACTGGCA
UTY	TCTAGGCTCTTTCATTATCATCCT[illegible]	AAGTCCTGAAGCAGAGGCAG
PPIE	TGTCCTCCCTGCAGATTCAC	GCACACAGTAGGAACCCCAG
NUDC	CTGGGAGAAGGGACAGCTTT	GCCCACAAGTCCCAGAGAAG
WDR77	GAGAGATGCGACTTGGTCCC	AGGCTCCTGTGTTGTCTCAC
SFPQ_1	GGAACTCCAGCAGGATATGGT	CCTGCCCAAACAGACCATTT
SFPQ_2	TGAGTGGTTCCATGATGGGA	ACCACACACCGAGTTCTTCT
NSL1	ACAGCTCTGAGTCTCAAGGC	GGTCACCCAGCTTCATCCTC
RABGGTB	TGGAATTGCTGGATTGTCACT	AGTCACTTTTAGATTCGATAAGCAC
ISG20L2	GCTTGCTCAGGTTGGGAAGA	AGCTGTCCATTGGTCCACTG
DTL	GCCCAGCTCCATGAGGAAAA	GGCAGCTTTCTTAAAGACCAGA
MAGOH	TGATGGGTCAAAGTTTTAATGCCT	ACACAAAATTTTCTACTCCCACCC
ZBTB8OS	TGTCACTAATCAGGATTGTGTGA	AAAAGTGTGGATGAGGCTGC
TRNAU1AP	GCTTGCTTTCCAGACCCCAT	GGAGTCTCTCATCTCCCTCAC
SNRPE	AAAACATCTGAGTGTGTGGCT	AGTAACACGAGGGTAATCAAAACA
MTOR	CCCTGAGCTTTCTAATTTGCCAC	GGCTGAGGTGGATGGATCAC
POLR1A	GGATCCCACGATGAGCTGAG	CACTTGGAACTGCCCTGGAT
ATL2	GCTCTTTCATCAGCACAGCG	TGGATGTGCAATTCCATACCCT
WDR33	TCTTTGTTTCAGGCCGAGGG	CCTCTACCCCAGTTACTGCC
UQCRC1	TGAGCAGCTCCCAGACTACA	GTGGCACAGGTTAGAGGAGC
HEK3	ATGTGGGCTGCCTAGAAAGG	CCCAGCCAAACTTGTCAACC
HEK2	CCAGCCCCATCTGTCAAACT	TGAATGGATTCCTTGGAAACAATGA
EMX1	CAGCTCAGCCTGAGTGTTGA	CTCGTGGGTTTGTGGTTGC
HEK4	GAACCCAGGTAGCCAGAGAC	TCCTTTCAACCCGAACGGAG
RNF2	ACGTCTCATATGCCCCTTGG	ACGTAGGAATTTTGGTGGGACA

Supplementary sequence. Full sequence of gBlock-PC plasmid.

Green label= hU6 promoter

Purple Label=gRNA scaffold

Grey Label= Ploy T+ linker

**TCGCGCGTTTCGGTGATGACGGTGAAAACCTCTGACACATGCAGCTCCCGGAGACGGTCAC
AGCTTGTCTGTAAGCGGATGCCGGGAGCAGACAAGCCCGTCAGGGCGCGTCAGCGGGTGT
TGGCGGGTGTCGGGGCTGGCTTAACTATGCGGCATCAGAGCAGATTGTACTGAGAGTGCAC
CATATGCGGTGTGAAATACCGCACAGATGCGTAAGGAGAAAATACCGCATCAGGCGCCATT
CGCCATTCAGGCTGCGCAACTGTTGGGAAGGGCGATCGGTGCGGGCCTCTTCGCTATTACG
CCAGCTGGCGAAAGGGGGATGTGCTGCAAGGCGATTAAGTTGGGTAACGCCAGGGTTTTC
CCAGTCACGACGTTGTAAAACGACGGCCAGTGAATTCGAGCTCGGTACCTCGCGAATGCAT
CTAGATGAAGACTGGCAAGAGGGCCTATTTCCCATGATTCCTTCATATTTGCATATACGATA
CAAGGCTGTTAGAGAGATAATTGGAATTAATTTGACTGTAAACACAAAGATATTAGTACAAA
ATACGTGACGTAGAAAGTAATAATTTCTTGGGTAGTTTGCAGTTTTAAAATTATGTTTTAAA
ATGGACTATCATATGCTTACCGTAACTTGAAAGTATTTCGATTTCTTGGCTTTATATATCTTG
TGGAAAGGACGAAACACCGGGCCCAGACTGAGCACGTGAGTTTTAGAGCTAGAAATAGCAA
GTTAAAATAAGGCTAGTCCGTTATCAACTTGAAAAAGTGGCACCGAGTCGGTGCTTTTTTGT
TTTAGAGCTAGAAATAGCAAGTTAAAATAAGGCTAGTCCGTTTTTAGCGCGTGCGCCAATTC
TGCAGACAAATGGCTCTAGAGAGGGCCTATTTCCCATGATTCCTTCATATTTGCATATACGA
TACAAGGCTGTTAGAGAGATAATTGGAATTAATTTGACTGTAAACACAAAGATATTAGTACA
AAATACGTGACGTAGAAAGTAATAATTTCTTGGGTAGTTTGCAGTTTTAAAATTATGTTTTA
AAATGGACTATCATATGCTTACCGTAACTTGAAAGTATTTCGATTTCTTGGCTTTATATATCT
TGTGGAAAGGACGAAACACCGGAACACAAAGCATAGACTGCGTTTTAGAGCTAGAAATAGC
AAGTTAAAATAAGGCTAGTCCGTTATCAACTTGAAAAAGTGGCACCGAGTCGGTGCTTTTTT
GTTTTAGAGCTAGAAATAGCAAGTTAAAATAAGGCTAGTCCGTTTTTAGCGCGTGCGCCAA
TTCTGCAGACAAATGGCTCTAGAGAGGGCCTATTTCCCATGATTCCTTCATATTTGCATATA
CGATACAAGGCTGTTAGAGAGATAATTGGAATTAATTTGACTGTAAACACAAAGATATTAGT
ACAAAATACGTGACGTAGAAAGTAATAATTTCTTGGGTAGTTTGCAGTTTTAAAATTATGTT
TTAAAATGGACTATCATATGCTTACCGTAACTTGAAAGTATTTCGATTTCTTGGCTTTATATA
TCTTGTGGAAAGGACGAAACACCGGAGTCCGAGCAGAAGAAGAAGTTTTAGAGCTAGAAAT
AGCAAGTTAAAATAAGGCTAGTCCGTTATCAACTTGAAAAAGTGGCACCGAGTCGGTGCTT
TTTTGTTTTAGAGCTAGAAATAGCAAGTTAAAATAAGGCTAGTCCGTTTTTAGCGCGTGCGC
CAATTCTGCAGACAAATGGCTCTAGAGAGGGCCTATTTCCCATGATTCCTTCATATTTGCAT
ATACGATACAAGGCTGTTAGAGAGATAATTGGAATTAATTTGACTGTAAACACAAAGATATT
AGTACAAAATACGTGACGTAGAAAGTAATAATTTCTTGGGTAGTTTGCAGTTTTAAAATTAT
GTTTTAAAATGGACTATCATATGCTTACCGTAACTTGAAAGTATTTCGATTTCTTGGCTTTAT
ATATCTTGTGGAAAGGACGAAACACCGGGCACTGCGGCTGGAGGTGGGTTTTAGAGCTAGA
AATAGCAAGTTAAAATAAGGCTAGTCCGTTATCAACTTGAAAAAGTGGCACCGAGTCGGTG
CTTTTTTGTTTTAGAGCTAGAAATAGCAAGTTAAAATAAGGCTAGTCCGTTTTTAGCGCGTG
CGCCAATTCTGCAGACAAATGGCTCTAGAGAGGGCCTATTTCCCATGATTCCTTCATATTTG
CATATACGATACAAGGCTGTTAGAGAGATAATTGGAATTAATTTGACTGTAAACACAAAGAT
ATTAGTACAAAATACGTGACGTAGAAAGTAATAATTTCTTGGGTAGTTTGCAGTTTTAAAAT
TATGTTTTAAAATGGACTATCATATGCTTACCGTAACTTGAAAGTATTTCGATTTCTTGGCTT
TATATATCTTGTGGAAAGGACGAAACACCGGTCATCTTAGTCATTACCTGGTTTTAGAGCTA
GAAATAGCAAGTTAAAATAAGGCTAGTCCGTTATCAACTTGAAAAAGTGGCACCGAGTCGG
TGCTTTTTTGTTTTAGAGCTAGAAATAGCAAGTTAAAATAAGGCTAGTCCGTTTTTAGCGCG**

**TGCGCCAATTCTGCAGACAAATGGCTCTAGAACTACAGTCTTCATCGGATCCCGGGCCCGT
CGACTGCAGAGGCCTGCATGCAAGCTTGGCGTAATCATGGTCATAGCTGTTTCCTGTGTGA
AATTGTTATCCGCTCACAATTCCACACAACATACGAGCCGGAAGCATAAAGTGTAAAGCCT
GGGGTGCCTAATGAGTGAGCTAACTCACATTAATTGCGTTGCGCTCACTGCCCGCTTTCCA
GTCGGGAAACCTGTCGTGCCAGCTGCATTAATGAATCGGCCAACGCGCGGGGAGAGGCGG
TTTGCGTATTGGGCGCTCTTCCGCTTCCTCGCTCACTGACTCGCTGCGCTCGGTCGTTCGG
CTGCGGCGAGCGGTATCAGCTCACTCAAAGGCGGTAATACGGTTATCCACAGAATCAGGGG
ATAACGCAGGAAAGAACATGTGAGCAAAAGGCCAGCAAAAGGCCAGGAACCGTAAAAAGG
CCGCGTTGCTGGCGTTTTTCCATAGGCTCCGCCCCCCTGACGAGCATCACAAAAATCGACG
CTCAAGTCAGAGGTGGCGAAACCCGACAGGACTATAAAGATACCAGGCGTTTCCCCCTGGA
AGCTCCCTCGTGCGCTCTCCTGTTCCGACCCTGCCGCTTACCGGATACCTGTCCGCCTTTCT
CCCTTCGGGAAGCGTGGCGCTTTCTCATAGCTCACGCTGTAGGTATCTCAGTTCGGTGTAG
GTCGTTCGCTCCAAGCTGGGCTGTGTGCACGAACCCCCCGTTCAGCCCGACCGCTGCGCCT
TATCCGGTAACTATCGTCTTGAGTCCAACCCGGTAAGACACGACTTATCGCCACTGGCAGC
AGCCACTGGTAACAGGATTAGCAGAGCGAGGTATGTAGGCGGTGCTACAGAGTTCTTGAAG
TGGTGGCCTAACTACGGCTACACTAGAAGAACAGTATTTGGTATCTGCGCTCTGCTGAAGC
CAGTTACCTTCGGAAAAAGAGTTGGTAGCTCTTGATCCGGCAAACAAACCACCGCTGGTAG
CGGTGGTTTTTTTGTTTGCAAGCAGCAGATTACGCGCAGAAAAAAAGGATCTCAAGAAGAT
CCTTTGATCTTTTCTACGGGGTCTGACGCTCAGTGGAACGAAAACTCACGTTAAGGGATTTT
GGTCATGAGATTATCAAAAAGGATCTTCACCTAGATCCTTTTAAATTAAAAATGAAGTTTTA
AATCAAGCCCAATCTGAATAATGTTACAACCAATTAACCAATTCTGATTAGAAAAACTCATC
GAGCATCAAATGAAACTGCAATTTATTCATATCAGGATTATCAATACCATATTTTTGAAAAA
GCCGTTTCTGTAATGAAGGAGAAAACTCACCGAGGCAGTTCCATAGGATGGCAAGATCCTG
GTATCGGTCTGCGATTCCGACTCGTCCAACATCAATACAACCTATTAATTTCCCCTCGTCAA
AAATAAGGTTATCAAGTGAGAAATCACCATGAGTGACGACTGAATCCGGTGAGAATGGCAA
AAGTTTATGCATTTCTTTCCAGACTTGTTCAACAGGCCAGCCATTACGCTCGTCATCAAAAT
CACTCGCATCAACCAAACCGTTATTCATTCGTGATTGCGCCTGAGCGAGACGAAATACGCG
ATCGCTGTTAAAAGGACAATTACAAACAGGAATCGAATGCAACCGGCGCAGGAACACTGCC
AGCGCATCAACAATATTTTCACCTGAATCAGGATATTCTTCTAATACCTGGAATGCTGTTTT
TCCGGGGATCGCAGTGGTGAGTAACCATGCATCATCAGGAGTACGGATAAAATGCTTGATG
GTCGGAAGAGGCATAAATTCCGTCAGCCAGTTTAGTCTGACCATCTCATCTGTAACATCATT
GGCAACGCTACCTTTGCCATGTTTCAGAAACAACTCTGGCGCATCGGGCTTCCCATACAAG
CGATAGATTGTCGCACCTGATTGCCCGACATTATCGCGAGCCCATTTATACCCATATAAATC
AGCATCCATGTTGGAATTTAATCGCGGCCTCGACGTTTCCCGTTGAATATGGCTCATAACAC
CCCTTGTATTACTGTTTATGTAAGCAGACAGTTTTATTGTTCATGATGATATATTTTTATCTT
GTGCAATGTAACATCAGAGATTTTGAGACACGGGCCAGAGCTGCA**

Sequence of 43-all-in-one plasmid

**CGTTACATAACTTACGGTAAATGGCCCGCCTGGCTGACCGCCCAACGACCCCCGCCCATTG
ACGTCAATAATGACGTATGTTCCCATAGTAACGCCAATAGGGACTTTCCATTGACGTCAATG
GGTGGAGTATTTACGGTAAACTGCCCACTTGGCAGTACATCAAGTGTATCATATGCCAAGT
ACGCCCCCTATTGACGTCAATGACGGTAAATGGCCCGCCTGGCATTATGCCCAGTACATGA
CCTTATGGGACTTTCCTACTTGGCAGTACATCTACGTATTAGTCATCGCTATTACCATGGTG
ATGCGGTTTTGGCAGTACATCAATGGGCGTGGATAGCGGTTTGACTCACGGGGATTTCCAA
GTCTCCACCCCATTGACGTCAATGGGAGTTTGTTTTGGCACCAAAATCAACGGGACTTTCCA
AAATGTCGTAACAACTCCGCCCCATTGACGCAAATGGGCGGTAGGCGTGTACGGTGGGAG
GTCTATATAAGCAGAGCTGGTTTAGTGAACCGTCAGATCCGCTAGCGCCACCATGGACAAC**

ACCGAGGACGTCATCAAGGAGTTCATGCAGTTCAAGGTGCGCATGGAGGGCTCCGTGAAC
GGCCACTACTTCGAGATCGAGGGCGAGGGCGAGGGCAAGCCCTACGAGGGCACCCAGACC
GCCAAGCTGCAGGTGACCAAGGGCGGCCCCCTGCCCTTCGCCTGGGACATCCTGTCCCCCC
AGTTCCAGTACGGCTCCAAGGCCTACGTGAAGCACCCCGCCGACATCCCCGACTACATGAA
GCTGTCCTTCCCCGAGGGCTTCACCTGGGAGCGCTCCATGAACTTCGAGGACGGCGGCGTG
GTGGAGGTGCAGCAGGACTCCTCCCTGCAGGACGGCACCTTCATCTACAAGGTGAAGTTCA
AGGGCGTGAACTTCCCCGCCGACGGCCCCGTAATGCAGAAGAAAACTGCCGGCTGGGAGC
CCTCCACCGAGAAGCTGTACCCCCAGGACGGCGTGCTGAAGGGCGAGATCTCCCACGCCCT
GAAGCTGAAGGACGGCGGCCACTACACCTGCGACTTCAAGACCGTGTACAAGGCCAAGAA
GCCCGTGCAGCTGCCCGGCAACCACTACGTGGACTCCAAGCTGGACATCACCAACCACAAC
GAGGACTACACCGTGGTGGAGCAGTACGAGCACGCCGAGGCCCGCCACTCCGGCTCCCAG
TAAGCGGCCGCACTCCTCAGGTGCAGGCTGCCTATCAGAAGGTGGTGGCTGGTGTGGCCA
ATGCCCTGGCTCACAAATACCACTGAGATCTTTTTCCCTCTGCCAAAAATTATGGGGACATC
ATGAAGCCCCTTGAGCATCTGACTTCTGGCTAATAAAGGAAATTTATTTTCATTGCAATAGT
GTGTTGGAATTTTTTGTGTCTCTCACTCGGAAGGACATATGGGAGGGCAAATCATTTAAAAC
ATCAGAATGAGTATTTGGTTTAGAGTTTGGCAACATATGCCCATATGCTGGCTGCCATGAAC
AAAGGTTGGCTATAAAGAGGTCATCAGTATATGAAACAGCCCCCTGCTGTCCATTCCTTATT
CCATAGAAAAGCCTTGACTTGAGGTTAGATTTTTTTTATATTTTGTTTTGTGTTATTTTTTTC
TTTAACATCCCTAAAATTTTCCTTACATGTTTTACTAGCCAGATTTTTCCTCCTCTCCTGACT
ACTCCCAGTCATAGCTGTCCCTCTTCTCTTATGGAGATCCCTCGACCTGCAGCCCAAGCTTG
GCGTAATCATGGTCATAGCTGTTTCCTGTGTGAAATTGTTATCCGCTCACAATTCCACACAA
CATACGAGCCGGAAGCATAAAGTGTAAAGCCTGGGGTGCCTAATGAGTGAGCTAACTCACA
TTAATTGCGTTGCGCTCACTGCCCGCTTTCCAGTCGGGAAACCTGTCGTGCCAGCGGATCC
GCATCTCAATTAGTCAGCAACCATAGTCCCGCCCCTAACTCCGCCCATCCCGCCCCTAACTC
CGCCCAGTTCCGCCCATTCTCCGCCCCATGGCTGACTAATTTTTTTTATTTATGCAGAGGCC
GAGGCCGCCTCGGCCTCTGAGCTATTCCAGAAGTAGTGAGGAGGCTTTTTTGGAGGCCTAG
GCTTTTGCAAAAAGCTAACTTGTTTATTGCAGCTTATAATGGTTACAAATAAAGCAATAGCA
TCACAAATTTCACAAATAAAGCATTTTTTTCACTGCATTCTAGTTGTGGTTTGTCCAAACTCA
TCAATGTATCTTATCATGTCTGGATCCGCTGCATTAATGAATCGGCCAACGCGCGGGGAGA
GGCGGTTTGCGTATTGGGCGCTCTTCCGCTTCCTCGCTCACTGACTCGCTGCGCTCGGTCG
TTCGGCTGCGGCGAGCGGTATCAGCTCACTCAAAGGCGGTAATACGGTTATCCACAGAATC
AGGGGATAACGCAGGAAAGAACATGTGAGCAAAAGGCCAGCAAAAGGCCAGGAACCGTAA
AAAGGCCGCGTTGCTGGCGTTTTTCCATAGGCTCCGCCCCCCTGACGAGCATCACAAAAAT
CGACGCTCAAGTCAGAGGTGGCGAAACCCGACAGGACTATAAAGATACCAGGCGTTTCCCC
CTGGAAGCTCCCTCGTGCGCTCTCCTGTTCCGACCCTGCCGCTTACCGGATACCTGTCCGC
CTTTCTCCCTTCGGGAAGCGTGGCGCTTTCTCATAGCTCACGCTGTAGGTATCTCAGTTCGG
TGTAGGTCGTTCGCTCCAAGCTGGGCTGTGTGCACGAACCCCCCGTTCAGCCCGACCGCTG
CGCCTTATCCGGTAACTATCGTCTTGAGTCCAACCCGGTAAGACACGACTTATCGCCACTG
GCAGCAGCCACTGGTAACAGGATTAGCAGAGCGAGGTATGTAGGCGGTGCTACAGAGTTCT
TGAAGTGGTGGCCTAACTACGGCTACACTAGAAGAACAGTATTTGGTATCTGCGCTCTGCT
GAAGCCAGTTACCTTCGGAAAAAGAGTTGGTAGCTCTTGATCCGGCAAACAAACCACCGCT
GGTAGCGGTGGTTTTTTTGTTTGCAAGCAGCAGATTACGCGCAGAAAAAAAGGATCTCAAG
AAGATCCTTTGATCTTTTCTACGGGGTCTGACGCTCAGTGGAACGAAAACTCACGTTAAGG
GATTTTGGTCATGAGATTATCAAAAAGGATCTTCACCTAGATCCTTTTAAATTAAAAATGAA
GTTTTAAATCAATCTAAAGTATATATGAGTAAACTTGGTCTGACAGTTACCAATGCTTAATC
AGTGAGGCACCTATCTCAGCGATCTGTCTATTTCGTTCATCCATAGTTGCCTGACTCCCCGT

CGTGTAGATAACTACGATACGGGAGGGCTTACCATCTGGCCCCAGTGCTGCAATGATACCG
CGAGACCCACGCTCACCGGCTCCAGATTTATCAGCAATAAACCAGCCAGCCGGAAGGGCCG
AGCGCAGAAGTGGTCCTGCAACTTTATCCGCCTCCATCCAGTCTATTAATTGTTGCCGGGA
AGCTAGAGTAAGTAGTTCGCCAGTTAATAGTTTGCGCAACGTTGTTGCCATTGCTACAGGC
ATCGTGGTGTCACGCTCGTCGTTTGGTATGGCTTCATTCAGCTCCGGTTCCCAACGATCAA
GGCGAGTTACATGATCCCCCATGTTGTGCAAAAAAGCGGTTAGCTCCTTCGGTCCTCCGAT
CGTTGTCAGAAGTAAGTTGGCCGCAGTGTTATCACTCATGGTTATGGCAGCACTGCATAAT
TCTCTTACTGTCATGCCATCCGTAAGATGCTTTTCTGTGACTGGTGAGTACTCAACCAAGTC
ATTCTGAGAATAGTGTATGCGGCGACCGAGTTGCTCTTGCCCGGCGTCAATACGGGATAAT
ACCGCGCCACATAGCAGAACTTTAAAAGTGCTCATCATTGGAAAACGTTCTTCGGGGCGAA
AACTCTCAAGGATCTTACCGCTGTTGAGATCCAGTTCGATGTAACCCACTCGTGCACCCAAC
TGATCTTCAGCATCTTTTACTTTCACCAGCGTTTCTGGGTGAGCAAAAACAGGAAGGCAAAA
TGCCGCAAAAAAGGGAATAAGGGCGACACGGAAATGTTGAATACTCATACTCTTCCTTTTT
CAATATTATTGAAGCATTTATCAGGGTTATTGTCTCATGAGCGGATACATATTTGAATGTAT
TTAGAAAAATAAACAAATAGGGGTTCCGCGCACATTTCCCCGAAAAGTGCCACCTGGGTCG
ACATTGATTATTGACTAGTTGCCGAGGGCCTATTTCCCATGATTCCTTCATATTTGCATATA
CGATACAAGGCTGTTAGAGAGATAATTGGAATTAATTTGACTGTAAACACAAAGATATTAGT
ACAAAATACGTGACGTAGAAAGTAATAATTTCTTGGGTAGTTTGCAGTTTTAAAATTATGTT
TTAAAATGGACTATCATATGCTTACCGTAACTTGAAAGTATTTCGATTTCTTGGCTTTATATA
TCTTGTGGAAAGGACGAAACACCGCCAAACCTAGCCTATTATCCGTTTTAGAGCTAGAAAT
AGCAAGTTAAAATAAGGCTAGTCCGTTATCAACTTGAAAAAGTGGCACCGAGTCGGTGCTT
TTTTGTTTTAGAGCTAGAAATAGCAAGTTAAAATAAGGCTAGTCCGTTTTTAGCGCGTGCGC
CAATTCTGCAGACAAATGGCTCTAGAGAGGGCCTATTTCCCATGATTCCTTCATATTTGCAT
ATACGATACAAGGCTGTTAGAGAGATAATTGGAATTAATTTGACTGTAAACACAAAGATATT
AGTACAAAATACGTGACGTAGAAAGTAATAATTTCTTGGGTAGTTTGCAGTTTTAAAATTAT
GTTTTAAAATGGACTATCATATGCTTACCGTAACTTGAAAGTATTTCGATTTCTTGGCTTTAT
ATATCTTGTGGAAAGGACGAAACACCGAGCTCTAATAAACCGAGCACGTTTTAGAGCTAGA
AATAGCAAGTTAAAATAAGGCTAGTCCGTTATCAACTTGAAAAAGTGGCACCGAGTCGGTG
CTTTTTTGTTTTAGAGCTAGAAATAGCAAGTTAAAATAAGGCTAGTCCGTTTTTAGCGCGTG
CGCCAATTCTGCAGACAAATGGCTCTAGAGAGGGCCTATTTCCCATGATTCCTTCATATTTG
CATATACGATACAAGGCTGTTAGAGAGATAATTGGAATTAATTTGACTGTAAACACAAAGAT
ATTAGTACAAAATACGTGACGTAGAAAGTAATAATTTCTTGGGTAGTTTGCAGTTTTAAAAT
TATGTTTTAAAATGGACTATCATATGCTTACCGTAACTTGAAAGTATTTCGATTTCTTGGCTT
TATATATCTTGTGGAAAGGACGAAACACCGCCCTCCTAGCCCGACGTGACGTTTTAGAGCT
AGAAATAGCAAGTTAAAATAAGGCTAGTCCGTTATCAACTTGAAAAAGTGGCACCGAGTCG
GTGCTTTTTTGTTTTAGAGCTAGAAATAGCAAGTTAAAATAAGGCTAGTCCGTTTTTAGCGC
GTGCGCCAATTCTGCAGACAAATGGCTCTAGAGAGGGCCTATTTCCCATGATTCCTTCATAT
TTGCATATACGATACAAGGCTGTTAGAGAGATAATTGGAATTAATTTGACTGTAAACACAAA
GATATTAGTACAAAATACGTGACGTAGAAAGTAATAATTTCTTGGGTAGTTTGCAGTTTTAA
AATTATGTTTTAAAATGGACTATCATATGCTTACCGTAACTTGAAAGTATTTCGATTTCTTG
GCTTTATATATCTTGTGGAAAGGACGAAACACCGGGCCCTAGGTGAGGATGTCAGTTTTAG
AGCTAGAAATAGCAAGTTAAAATAAGGCTAGTCCGTTATCAACTTGAAAAAGTGGCACCGA
GTCGGTGCTTTTTTGTTTTAGAGCTAGAAATAGCAAGTTAAAATAAGGCTAGTCCGTTTTTA
GCGCGTGCGCCAATTCTGCAGACAAATGGCTCTAGAGAGGGCCTATTTCCCATGATTCCTT
CATATTTGCATATACGATACAAGGCTGTTAGAGAGATAATTGGAATTAATTTGACTGTAAAC
ACAAAGATATTAGTACAAAATACGTGACGTAGAAAGTAATAATTTCTTGGGTAGTTTGCAGT

TTTAAAATTATGTTTTAAAATGGACTATCATATGCTTACCGTAACTTGAAAGTATTTCGATTT
CTTGGCTTTATATATCTTGTGGAAAGGACGAAACACCGCCATCTAAGATAGCAGCAGCGTT
TTAGAGCTAGAAATAGCAAGTTAAAATAAGGCTAGTCCGTTATCAACTTGAAAAAGTGGCA
CCGAGTCGGTGCTTTTTTGTTTTAGAGCTAGAAATAGCAAGTTAAAATAAGGCTAGTCCGTT
TTTAGCGCGTGCGCCAATTCTGCAGACAAATGGCTCTAGAGCAAGAGGGCCTATTTCCCAT
GATTCCTTCATATTTGCATATACGATACAAGGCTGTTAGAGAGATAATTGGAATTAATTTGA
CTGTAAACACAAAGATATTAGTACAAAATACGTGACGTAGAAAGTAATAATTTCTTGGGTAG
TTTGCAGTTTTAAAATTATGTTTTAAAATGGACTATCATATGCTTACCGTAACTTGAAAGTAT
TTCGATTTCTTGGCTTTATATATCTTGTGGAAAGGACGAAACACCGCCTAGCTACTTGGGAG
TCTGGTTTTAGAGCTAGAAATAGCAAGTTAAAATAAGGCTAGTCCGTTATCAACTTGAAAAA
GTGGCACCGAGTCGGTGCTTTTTTGTTTTAGAGCTAGAAATAGCAAGTTAAAATAAGGCTA
GTCCGTTTTTAGCGCGTGCGCCAATTCTGCAGACAAATGGCTCTAGAGAGGGCCTATTTCC
CATGATTCCTTCATATTTGCATATACGATACAAGGCTGTTAGAGAGATAATTGGAATTAATT
TGACTGTAAACACAAAGATATTAGTACAAAATACGTGACGTAGAAAGTAATAATTTCTTGGG
TAGTTTGCAGTTTTAAAATTATGTTTTAAAATGGACTATCATATGCTTACCGTAACTTGAAA
GTATTTCGATTTCTTGGCTTTATATATCTTGTGGAAAGGACGAAACACCGTCTCTAGAGATG
GTTTATCAGTTTTAGAGCTAGAAATAGCAAGTTAAAATAAGGCTAGTCCGTTATCAACTTGA
AAAAGTGGCACCGAGTCGGTGCTTTTTTGTTTTAGAGCTAGAAATAGCAAGTTAAAATAAG
GCTAGTCCGTTTTTAGCGCGTGCGCCAATTCTGCAGACAAATGGCTCTAGAGAGGGCCTAT
TTCCCATGATTCCTTCATATTTGCATATACGATACAAGGCTGTTAGAGAGATAATTGGAATT
AATTTGACTGTAAACACAAAGATATTAGTACAAAATACGTGACGTAGAAAGTAATAATTTCT
TGGGTAGTTTGCAGTTTTAAAATTATGTTTTAAAATGGACTATCATATGCTTACCGTAACTT
GAAAGTATTTCGATTTCTTGGCTTTATATATCTTGTGGAAAGGACGAAACACCGAGAATCTC
TATGTCTTTTGGGTTTTAGAGCTAGAAATAGCAAGTTAAAATAAGGCTAGTCCGTTATCAAC
TTGAAAAAGTGGCACCGAGTCGGTGCTTTTTTGTTTTAGAGCTAGAAATAGCAAGTTAAAAT
AAGGCTAGTCCGTTTTTAGCGCGTGCGCCAATTCTGCAGACAAATGGCTCTAGAGAGGGCC
TATTTCCCATGATTCCTTCATATTTGCATATACGATACAAGGCTGTTAGAGAGATAATTGGA
ATTAATTTGACTGTAAACACAAAGATATTAGTACAAAATACGTGACGTAGAAAGTAATAATT
TCTTGGGTAGTTTGCAGTTTTAAAATTATGTTTTAAAATGGACTATCATATGCTTACCGTAA
CTTGAAAGTATTTCGATTTCTTGGCTTTATATATCTTGTGGAAAGGACGAAACACCGTTTGG
CTACTTGGTCTCTTCGTTTTAGAGCTAGAAATAGCAAGTTAAAATAAGGCTAGTCCGTTATC
AACTTGAAAAAGTGGCACCGAGTCGGTGCTTTTTTGTTTTAGAGCTAGAAATAGCAAGTTAA
AATAAGGCTAGTCCGTTTTTAGCGCGTGCGCCAATTCTGCAGACAAATGGCTCTAGAGAGG
GCCTATTTCCCATGATTCCTTCATATTTGCATATACGATACAAGGCTGTTAGAGAGATAATT
GGAATTAATTTGACTGTAAACACAAAGATATTAGTACAAAATACGTGACGTAGAAAGTAATA
ATTTCTTGGGTAGTTTGCAGTTTTAAAATTATGTTTTAAAATGGACTATCATATGCTTACCGT
AACTTGAAAGTATTTCGATTTCTTGGCTTTATATATCTTGTGGAAAGGACGAAACACCGGAT
GCTTCTAGAAGCCTGGAGTTTTAGAGCTAGAAATAGCAAGTTAAAATAAGGCTAGTCCGTT
ATCAACTTGAAAAAGTGGCACCGAGTCGGTGCTTTTTTGTTTTAGAGCTAGAAATAGCAAGT
TAAAATAAGGCTAGTCCGTTTTTAGCGCGTGCGCCAATTCTGCAGACAAATGGCTCTAGAA
CTAGAGGGCCTATTTCCCATGATTCCTTCATATTTGCATATACGATACAAGGCTGTTAGAGA
GATAATTGGAATTAATTTGACTGTAAACACAAAGATATTAGTACAAAATACGTGACGTAGAA
AGTAATAATTTCTTGGGTAGTTTGCAGTTTTAAAATTATGTTTTAAAATGGACTATCATATG
CTTACCGTAACTTGAAAGTATTTCGATTTCTTGGCTTTATATATCTTGTGGAAAGGACGAAA
CACCGTTCGTCCCTAGCTCTGAAGGGTTTTAGAGCTAGAAATAGCAAGTTAAAATAAGGCT
AGTCCGTTATCAACTTGAAAAAGTGGCACCGAGTCGGTGCTTTTTTGTTTTAGAGCTAGAAA

TAGCAAGTTAAAATAAGGCTAGTCCGTTTTTAGCGCGTGCGCCAATTCTGCAGACAAATGG
CTCTAGAGAGGGCCTATTTCCCATGATTCCTTCATATTTGCATATACGATACAAGGCTGTTA
GAGAGATAATTGGAATTAATTTGACTGTAAACACAAAGATATTAGTACAAAATACGTGACGT
AGAAAGTAATAATTTCTTGGGTAGTTTGCAGTTTTAAAATTATGTTTTAAAATGGACTATCA
TATGCTTACCGTAACTTGAAAGTATTTCGATTTCTTGGCTTTATATATCTTGTGGAAAGGAC
GAAACACCGCCTTTCCTAAGACATTGCTAGTTTTAGAGCTAGAAATAGCAAGTTAAAATAAG
GCTAGTCCGTTATCAACTTGAAAAAGTGGCACCGAGTCGGTGCTTTTTTGTTTTAGAGCTAG
AAATAGCAAGTTAAAATAAGGCTAGTCCGTTTTTAGCGCGTGCGCCAATTCTGCAGACAAA
TGGCTCTAGAGAGGGCCTATTTCCCATGATTCCTTCATATTTGCATATACGATACAAGGCTG
TTAGAGAGATAATTGGAATTAATTTGACTGTAAACACAAAGATATTAGTACAAAATACGTGA
CGTAGAAAGTAATAATTTCTTGGGTAGTTTGCAGTTTTAAAATTATGTTTTAAAATGGACTA
TCATATGCTTACCGTAACTTGAAAGTATTTCGATTTCTTGGCTTTATATATCTTGTGGAAAG
GACGAAACACCGTGGAGGCTACTCCAGAACCAGTTTTAGAGCTAGAAATAGCAAGTTAAAA
TAAGGCTAGTCCGTTATCAACTTGAAAAAGTGGCACCGAGTCGGTGCTTTTTTGTTTTAGAG
CTAGAAATAGCAAGTTAAAATAAGGCTAGTCCGTTTTTAGCGCGTGCGCCAATTCTGCAGA
CAAATGGCTCTAGAGAGGGCCTATTTCCCATGATTCCTTCATATTTGCATATACGATACAAG
GCTGTTAGAGAGATAATTGGAATTAATTTGACTGTAAACACAAAGATATTAGTACAAAATAC
GTGACGTAGAAAGTAATAATTTCTTGGGTAGTTTGCAGTTTTAAAATTATGTTTTAAAATGG
ACTATCATATGCTTACCGTAACTTGAAAGTATTTCGATTTCTTGGCTTTATATATCTTGTGGA
AAGGACGAAACACCGGAAAAGCTATACTCTAGTGGGTTTTAGAGCTAGAAATAGCAAGTTA
AAATAAGGCTAGTCCGTTATCAACTTGAAAAAGTGGCACCGAGTCGGTGCTTTTTTGTTTTA
GAGCTAGAAATAGCAAGTTAAAATAAGGCTAGTCCGTTTTTAGCGCGTGCGCCAATTCTGC
AGACAAATGGCTCTAGAGAGGGCCTATTTCCCATGATTCCTTCATATTTGCATATACGATAC
AAGGCTGTTAGAGAGATAATTGGAATTAATTTGACTGTAAACACAAAGATATTAGTACAAAA
TACGTGACGTAGAAAGTAATAATTTCTTGGGTAGTTTGCAGTTTTAAAATTATGTTTTAAAA
TGGACTATCATATGCTTACCGTAACTTGAAAGTATTTCGATTTCTTGGCTTTATATATCTTGT
GGAAAGGACGAAACACCGTGTCCTACAGCTTTGTCCAGGTTTTAGAGCTAGAAATAGCAAG
TTAAAATAAGGCTAGTCCGTTATCAACTTGAAAAAGTGGCACCGAGTCGGTGCTTTTTTGTT
TTAGAGCTAGAAATAGCAAGTTAAAATAAGGCTAGTCCGTTTTTAGCGCGTGCGCCAATTCT
GCAGACAAATGGCTCTAGATTACGAGGGCCTATTTCCCATGATTCCTTCATATTTGCATATA
CGATACAAGGCTGTTAGAGAGATAATTGGAATTAATTTGACTGTAAACACAAAGATATTAGT
ACAAAATACGTGACGTAGAAAGTAATAATTTCTTGGGTAGTTTGCAGTTTTAAAATTATGTT
TTAAAATGGACTATCATATGCTTACCGTAACTTGAAAGTATTTCGATTTCTTGGCTTTATATA
TCTTGTGGAAAGGACGAAACACCGTTCAGTTCTAAGTCAACGTTGTTTTAGAGCTAGAAATA
GCAAGTTAAAATAAGGCTAGTCCGTTATCAACTTGAAAAAGTGGCACCGAGTCGGTGCTTT
TTTGTTTTAGAGCTAGAAATAGCAAGTTAAAATAAGGCTAGTCCGTTTTTAGCGCGTGCGCC
AATTCTGCAGACAAATGGCTCTAGAGAGGGCCTATTTCCCATGATTCCTTCATATTTGCATA
TACGATACAAGGCTGTTAGAGAGATAATTGGAATTAATTTGACTGTAAACACAAAGATATTA
GTACAAAATACGTGACGTAGAAAGTAATAATTTCTTGGGTAGTTTGCAGTTTTAAAATTATG
TTTTAAAATGGACTATCATATGCTTACCGTAACTTGAAAGTATTTCGATTTCTTGGCTTTATA
TATCTTGTGGAAAGGACGAAACACCGATCTCCTAATTCTTCACAAAGTTTTAGAGCTAGAAA
TAGCAAGTTAAAATAAGGCTAGTCCGTTATCAACTTGAAAAAGTGGCACCGAGTCGGTGCT
TTTTTGTTTTAGAGCTAGAAATAGCAAGTTAAAATAAGGCTAGTCCGTTTTTAGCGCGTGCG
CCAATTCTGCAGACAAATGGCTCTAGAGAGGGCCTATTTCCCATGATTCCTTCATATTTGCA
TATACGATACAAGGCTGTTAGAGAGATAATTGGAATTAATTTGACTGTAAACACAAAGATAT
TAGTACAAAATACGTGACGTAGAAAGTAATAATTTCTTGGGTAGTTTGCAGTTTTAAAATTA

TGTTTTAAAATGGACTATCATATGCTTACCGTAACTTGAAAGTATTTCGATTTCTTGGCTTTA
TATATCTTGTGGAAAGGACGAAACACCGTGCCTACTCATTTTCTTCACGTTTTAGAGCTAGA
AATAGCAAGTTAAAATAAGGCTAGTCCGTTATCAACTTGAAAAAGTGGCACCGAGTCGGTG
CTTTTTTGTTTTAGAGCTAGAAATAGCAAGTTAAAATAAGGCTAGTCCGTTTTTAGCGCGTG
CGCCAATTCTGCAGACAAATGGCTCTAGAGAGGGCCTATTTCCCATGATTCCTTCATATTTG
CATATACGATACAAGGCTGTTAGAGAGATAATTGGAATTAATTTGACTGTAAACACAAAGAT
ATTAGTACAAAATACGTGACGTAGAAAGTAATAATTTCTTGGGTAGTTTGCAGTTTTAAAAT
TATGTTTTAAAATGGACTATCATATGCTTACCGTAACTTGAAAGTATTTCGATTTCTTGGCTT
TATATATCTTGTGGAAAGGACGAAACACCGATGGTGGCTATCCACGATGGGTTTTAGAGCT
AGAAATAGCAAGTTAAAATAAGGCTAGTCCGTTATCAACTTGAAAAAGTGGCACCGAGTCG
GTGCTTTTTTGTTTTAGAGCTAGAAATAGCAAGTTAAAATAAGGCTAGTCCGTTTTTAGCGC
GTGCGCCAATTCTGCAGACAAATGGCTCTAGAGAGGGCCTATTTCCCATGATTCCTTCATAT
TTGCATATACGATACAAGGCTGTTAGAGAGATAATTGGAATTAATTTGACTGTAAACACAAA
GATATTAGTACAAAATACGTGACGTAGAAAGTAATAATTTCTTGGGTAGTTTGCAGTTTTAA
AATTATGTTTTAAAATGGACTATCATATGCTTACCGTAACTTGAAAGTATTTCGATTTCTTG
GCTTTATATATCTTGTGGAAAGGACGAAACACCGATAGCTAAACACTCATCATTGTTTTAGA
GCTAGAAATAGCAAGTTAAAATAAGGCTAGTCCGTTATCAACTTGAAAAAGTGGCACCGAG
TCGGTGCTTTTTTGTTTTAGAGCTAGAAATAGCAAGTTAAAATAAGGCTAGTCCGTTTTTAG
CGCGTGCGCCAATTCTGCAGACAAATGGCTCTAGATGTGGAGGGCCTATTTCCCATGATTC
CTTCATATTTGCATATACGATACAAGGCTGTTAGAGAGATAATTGGAATTAATTTGACTGTA
AACACAAAGATATTAGTACAAAATACGTGACGTAGAAAGTAATAATTTCTTGGGTAGTTTGC
AGTTTTAAAATTATGTTTTAAAATGGACTATCATATGCTTACCGTAACTTGAAAGTATTTCG
ATTTCTTGGCTTTATATATCTTGTGGAAAGGACGAAACACCGGCCAACTATGGCGTGACAG
AGTTTTAGAGCTAGAAATAGCAAGTTAAAATAAGGCTAGTCCGTTATCAACTTGAAAAAGTG
GCACCGAGTCGGTGCTTTTTTGTTTTAGAGCTAGAAATAGCAAGTTAAAATAAGGCTAGTCC
GTTTTTAGCGCGTGCGCCAATTCTGCAGACAAATGGCTCTAGAGAGGGCCTATTTCCCATG
ATTCCTTCATATTTGCATATACGATACAAGGCTGTTAGAGAGATAATTGGAATTAATTTGAC
TGTAAACACAAAGATATTAGTACAAAATACGTGACGTAGAAAGTAATAATTTCTTGGGTAGT
TTGCAGTTTTAAAATTATGTTTTAAAATGGACTATCATATGCTTACCGTAACTTGAAAGTATT
TCGATTTCTTGGCTTTATATATCTTGTGGAAAGGACGAAACACCGTCATTCTATTTGCCTTT
TTTGTTTTAGAGCTAGAAATAGCAAGTTAAAATAAGGCTAGTCCGTTATCAACTTGAAAAAG
TGGCACCGAGTCGGTGCTTTTTTGTTTTAGAGCTAGAAATAGCAAGTTAAAATAAGGCTAGT
CCGTTTTTAGCGCGTGCGCCAATTCTGCAGACAAATGGCTCTAGAGAGGGCCTATTTCCCA
TGATTCCTTCATATTTGCATATACGATACAAGGCTGTTAGAGAGATAATTGGAATTAATTTG
ACTGTAAACACAAAGATATTAGTACAAAATACGTGACGTAGAAAGTAATAATTTCTTGGGTA
GTTTGCAGTTTTAAAATTATGTTTTAAAATGGACTATCATATGCTTACCGTAACTTGAAAGT
ATTTCGATTTCTTGGCTTTATATATCTTGTGGAAAGGACGAAACACCGGCTTTCTAGCAGCC
TCCCCAGTTTTAGAGCTAGAAATAGCAAGTTAAAATAAGGCTAGTCCGTTATCAACTTGAAA
AAGTGGCACCGAGTCGGTGCTTTTTTGTTTTAGAGCTAGAAATAGCAAGTTAAAATAAGGC
TAGTCCGTTTTTAGCGCGTGCGCCAATTCTGCAGACAAATGGCTCTAGAGAGGGCCTATTT
CCCATGATTCCTTCATATTTGCATATACGATACAAGGCTGTTAGAGAGATAATTGGAATTAA
TTTGACTGTAAACACAAAGATATTAGTACAAAATACGTGACGTAGAAAGTAATAATTTCTTG
GGTAGTTTGCAGTTTTAAAATTATGTTTTAAAATGGACTATCATATGCTTACCGTAACTTGA
AAGTATTTCGATTTCTTGGCTTTATATATCTTGTGGAAAGGACGAAACACCGTTGTGCTACA
GACTAAATCCGTTTTAGAGCTAGAAATAGCAAGTTAAAATAAGGCTAGTCCGTTATCAACTT
GAAAAAGTGGCACCGAGTCGGTGCTTTTTTGTTTTAGAGCTAGAAATAGCAAGTTAAAATA

AGGCTAGTCCGTTTTTAGCGCGTGCGCCAATTCTGCAGACAAATGGCTCTAGAGAGGGCCT
ATTTCCCATGATTCCTTCATATTTGCATATACGATACAAGGCTGTTAGAGAGATAATTGGAA
TTAATTTGACTGTAAACACAAAGATATTAGTACAAAATACGTGACGTAGAAAGTAATAATTT
CTTGGGTAGTTTGCAGTTTTAAAATTATGTTTTAAAATGGACTATCATATGCTTACCGTAAC
TTGAAAGTATTTCGATTTCTTGGCTTTATATATCTTGTGGAAAGGACGAAACACCGACAACT
AAAGTTCTGAGCTAGTTTTAGAGCTAGAAATAGCAAGTTAAAATAAGGCTAGTCCGTTATCA
ACTTGAAAAAGTGGCACCGAGTCGGTGCTTTTTTGTTTTAGAGCTAGAAATAGCAAGTTAAA
ATAAGGCTAGTCCGTTTTTAGCGCGTGCGCCAATTCTGCAGACAAATGGCTCTAGAGAGCG
AGGGCCTATTTCCCATGATTCCTTCATATTTGCATATACGATACAAGGCTGTTAGAGAGATA
ATTGGAATTAATTTGACTGTAAACACAAAGATATTAGTACAAAATACGTGACGTAGAAAGTA
ATAATTTCTTGGGTAGTTTGCAGTTTTAAAATTATGTTTTAAAATGGACTATCATATGCTTAC
CGTAACTTGAAAGTATTTCGATTTCTTGGCTTTATATATCTTGTGGAAAGGACGAAACACCG
GATTCCTAAGACTGTTTGCTGTTTTAGAGCTAGAAATAGCAAGTTAAAATAAGGCTAGTCCG
TTATCAACTTGAAAAAGTGGCACCGAGTCGGTGCTTTTTTGTTTTAGAGCTAGAAATAGCAA
GTTAAAATAAGGCTAGTCCGTTTTTAGCGCGTGCGCCAATTCTGCAGACAAATGGCTCTAG
AGAGGGCCTATTTCCCATGATTCCTTCATATTTGCATATACGATACAAGGCTGTTAGAGAGA
TAATTGGAATTAATTTGACTGTAAACACAAAGATATTAGTACAAAATACGTGACGTAGAAAG
TAATAATTTCTTGGGTAGTTTGCAGTTTTAAAATTATGTTTTAAAATGGACTATCATATGCTT
ACCGTAACTTGAAAGTATTTCGATTTCTTGGCTTTATATATCTTGTGGAAAGGACGAAACAC
CGCTTTTCCTAGTGCCCATACCGTTTTAGAGCTAGAAATAGCAAGTTAAAATAAGGCTAGTC
CGTTATCAACTTGAAAAAGTGGCACCGAGTCGGTGCTTTTTTGTTTTAGAGCTAGAAATAGC
AAGTTAAAATAAGGCTAGTCCGTTTTTAGCGCGTGCGCCAATTCTGCAGACAAATGGCTCT
AGAGAGGGCCTATTTCCCATGATTCCTTCATATTTGCATATACGATACAAGGCTGTTAGAGA
GATAATTGGAATTAATTTGACTGTAAACACAAAGATATTAGTACAAAATACGTGACGTAGAA
AGTAATAATTTCTTGGGTAGTTTGCAGTTTTAAAATTATGTTTTAAAATGGACTATCATATG
CTTACCGTAACTTGAAAGTATTTCGATTTCTTGGCTTTATATATCTTGTGGAAAGGACGAAA
CACCGCAAGGCTAAGTCCCACGTGCGTTTTAGAGCTAGAAATAGCAAGTTAAAATAAGGCT
AGTCCGTTATCAACTTGAAAAAGTGGCACCGAGTCGGTGCTTTTTTGTTTTAGAGCTAGAAA
TAGCAAGTTAAAATAAGGCTAGTCCGTTTTTAGCGCGTGCGCCAATTCTGCAGACAAATGG
CTCTAGAGAGGGCCTATTTCCCATGATTCCTTCATATTTGCATATACGATACAAGGCTGTTA
GAGAGATAATTGGAATTAATTTGACTGTAAACACAAAGATATTAGTACAAAATACGTGACGT
AGAAAGTAATAATTTCTTGGGTAGTTTGCAGTTTTAAAATTATGTTTTAAAATGGACTATCA
TATGCTTACCGTAACTTGAAAGTATTTCGATTTCTTGGCTTTATATATCTTGTGGAAAGGAC
GAAACACCGCAATCTTCTATTTCTCTAAAGTTTTAGAGCTAGAAATAGCAAGTTAAAATAAG
GCTAGTCCGTTATCAACTTGAAAAAGTGGCACCGAGTCGGTGCTTTTTTGTTTTAGAGCTAG
AAATAGCAAGTTAAAATAAGGCTAGTCCGTTTTTAGCGCGTGCGCCAATTCTGCAGACAAA
TGGCTCTAGAGAGGGCCTATTTCCCATGATTCCTTCATATTTGCATATACGATACAAGGCTG
TTAGAGAGATAATTGGAATTAATTTGACTGTAAACACAAAGATATTAGTACAAAATACGTGA
CGTAGAAAGTAATAATTTCTTGGGTAGTTTGCAGTTTTAAAATTATGTTTTAAAATGGACTA
TCATATGCTTACCGTAACTTGAAAGTATTTCGATTTCTTGGCTTTATATATCTTGTGGAAAG
GACGAAACACCGTCCTCGCTACAGGAAGCTGCGTTTTAGAGCTAGAAATAGCAAGTTAAAA
TAAGGCTAGTCCGTTATCAACTTGAAAAAGTGGCACCGAGTCGGTGCTTTTTTGTTTTAGAG
CTAGAAATAGCAAGTTAAAATAAGGCTAGTCCGTTTTTAGCGCGTGCGCCAATTCTGCAGA
CAAATGGCTCTAGAAGGAGAGGGCCTATTTCCCATGATTCCTTCATATTTGCATATACGATA
CAAGGCTGTTAGAGAGATAATTGGAATTAATTTGACTGTAAACACAAAGATATTAGTACAAA
ATACGTGACGTAGAAAGTAATAATTTCTTGGGTAGTTTGCAGTTTTAAAATTATGTTTTAAA

ATGGACTATCATATGCTTACCGTAACTTGAAAGTATTTCGATTTCTTGGCTTTATATATCTTG
TGGAAAGGACGAAACACCGTCTTTCCTATTTCTTCACACGTTTTAGAGCTAGAAATAGCAAG
TTAAAATAAGGCTAGTCCGTTATCAACTTGAAAAAGTGGCACCGAGTCGGTGCTTTTTTGTT
TTAGAGCTAGAAATAGCAAGTTAAAATAAGGCTAGTCCGTTTTTAGCGCGTGCGCCAATTCT
GCAGACAAATGGCTCTAGAGAGGGCCTATTTCCCATGATTCCTTCATATTTGCATATACGAT
ACAAGGCTGTTAGAGAGATAATTGGAATTAATTTGACTGTAAACACAAAGATATTAGTACAA
AATACGTGACGTAGAAAGTAATAATTTCTTGGGTAGTTTGCAGTTTTAAAATTATGTTTTAA
AATGGACTATCATATGCTTACCGTAACTTGAAAGTATTTCGATTTCTTGGCTTTATATATCTT
GTGGAAAGGACGAAACACCGGAAACAGCTACAAAACCAGTGTTTTAGAGCTAGAAATAGCA
AGTTAAAATAAGGCTAGTCCGTTATCAACTTGAAAAAGTGGCACCGAGTCGGTGCTTTTTTG
TTTTAGAGCTAGAAATAGCAAGTTAAAATAAGGCTAGTCCGTTTTTAGCGCGTGCGCCAATT
CTGCAGACAAATGGCTCTAGAGAGGGCCTATTTCCCATGATTCCTTCATATTTGCATATACG
ATACAAGGCTGTTAGAGAGATAATTGGAATTAATTTGACTGTAAACACAAAGATATTAGTAC
AAAATACGTGACGTAGAAAGTAATAATTTCTTGGGTAGTTTGCAGTTTTAAAATTATGTTTT
AAAATGGACTATCATATGCTTACCGTAACTTGAAAGTATTTCGATTTCTTGGCTTTATATAT
CTTGTGGAAAGGACGAAACACCGGAGCTCTACGTCAGCTTCCAGTTTTAGAGCTAGAAATA
GCAAGTTAAAATAAGGCTAGTCCGTTATCAACTTGAAAAAGTGGCACCGAGTCGGTGCTTT
TTTGTTTTAGAGCTAGAAATAGCAAGTTAAAATAAGGCTAGTCCGTTTTTAGCGCGTGCGCC
AATTCTGCAGACAAATGGCTCTAGAGAGGGCCTATTTCCCATGATTCCTTCATATTTGCATA
TACGATACAAGGCTGTTAGAGAGATAATTGGAATTAATTTGACTGTAAACACAAAGATATTA
GTACAAAATACGTGACGTAGAAAGTAATAATTTCTTGGGTAGTTTGCAGTTTTAAAATTATG
TTTTAAAATGGACTATCATATGCTTACCGTAACTTGAAAGTATTTCGATTTCTTGGCTTTATA
TATCTTGTGGAAAGGACGAAACACCGGGGCTAGTTGAATTTAGCCTGTTTTAGAGCTAGAA
ATAGCAAGTTAAAATAAGGCTAGTCCGTTATCAACTTGAAAAAGTGGCACCGAGTCGGTGC
TTTTTTGTTTTAGAGCTAGAAATAGCAAGTTAAAATAAGGCTAGTCCGTTTTTAGCGCGTGC
GCCAATTCTGCAGACAAATGGCTCTAGAGAGGGCCTATTTCCCATGATTCCTTCATATTTGC
ATATACGATACAAGGCTGTTAGAGAGATAATTGGAATTAATTTGACTGTAAACACAAAGATA
TTAGTACAAAATACGTGACGTAGAAAGTAATAATTTCTTGGGTAGTTTGCAGTTTTAAAATT
ATGTTTTAAAATGGACTATCATATGCTTACCGTAACTTGAAAGTATTTCGATTTCTTGGCTTT
ATATATCTTGTGGAAAGGACGAAACACCGCCAATCTACTCAGTAACACTGTTTTAGAGCTAG
AAATAGCAAGTTAAAATAAGGCTAGTCCGTTATCAACTTGAAAAAGTGGCACCGAGTCGGT
GCTTTTTTGTTTTAGAGCTAGAAATAGCAAGTTAAAATAAGGCTAGTCCGTTTTTAGCGCGT
GCGCCAATTCTGCAGACAAATGGCTCTAGAATTCGAGGGCCTATTTCCCATGATTCCTTCAT
ATTTGCATATACGATACAAGGCTGTTAGAGAGATAATTGGAATTAATTTGACTGTAAACACA
AAGATATTAGTACAAAATACGTGACGTAGAAAGTAATAATTTCTTGGGTAGTTTGCAGTTTT
AAAATTATGTTTTAAAATGGACTATCATATGCTTACCGTAACTTGAAAGTATTTCGATTTCTT
GGCTTTATATATCTTGTGGAAAGGACGAAACACCGCATCTAAAATCGGGGTTTTTGTTTTAG
AGCTAGAAATAGCAAGTTAAAATAAGGCTAGTCCGTTATCAACTTGAAAAAGTGGCACCGA
GTCGGTGCTTTTTTGTTTTAGAGCTAGAAATAGCAAGTTAAAATAAGGCTAGTCCGTTTTTA
GCGCGTGCGCCAATTCTGCAGACAAATGGCTCTAGAGAGGGCCTATTTCCCATGATTCCTT
CATATTTGCATATACGATACAAGGCTGTTAGAGAGATAATTGGAATTAATTTGACTGTAAAC
ACAAAGATATTAGTACAAAATACGTGACGTAGAAAGTAATAATTTCTTGGGTAGTTTGCAGT
TTTAAAATTATGTTTTAAAATGGACTATCATATGCTTACCGTAACTTGAAAGTATTTCGATTT
CTTGGCTTTATATATCTTGTGGAAAGGACGAAACACCGACACACCTAAGTTGTGAAAAGTTT
TAGAGCTAGAAATAGCAAGTTAAAATAAGGCTAGTCCGTTATCAACTTGAAAAAGTGGCAC
CGAGTCGGTGCTTTTTTGTTTTAGAGCTAGAAATAGCAAGTTAAAATAAGGCTAGTCCGTTT

TTAGCGCGTGCGCCAATTCTGCAGACAAATGGCTCTAGAGAGGGCCTATTTCCCATGATTC
CTTCATATTTGCATATACGATACAAGGCTGTTAGAGAGATAATTGGAATTAATTTGACTGTA
AACACAAAGATATTAGTACAAAATACGTGACGTAGAAAGTAATAATTTCTTGGGTAGTTTGC
AGTTTTAAAATTATGTTTTAAAATGGACTATCATATGCTTACCGTAACTTGAAAGTATTTCG
ATTTCTTGGCTTTATATATCTTGTGGAAAGGACGAAACACCGCTCTCCTAAACTGCCCCTAG
GTTTTAGAGCTAGAAATAGCAAGTTAAAATAAGGCTAGTCCGTTATCAACTTGAAAAAGTG
GCACCGAGTCGGTGCTTTTTTGTTTTAGAGCTAGAAATAGCAAGTTAAAATAAGGCTAGTCC
GTTTTTAGCGCGTGCGCCAATTCTGCAGACAAATGGCTCTAGAGAGGGCCTATTTCCCATG
ATTCCTTCATATTTGCATATACGATACAAGGCTGTTAGAGAGATAATTGGAATTAATTTGAC
TGTAAACACAAAGATATTAGTACAAAATACGTGACGTAGAAAGTAATAATTTCTTGGGTAGT
TTGCAGTTTTAAAATTATGTTTTAAAATGGACTATCATATGCTTACCGTAACTTGAAAGTATT
TCGATTTCTTGGCTTTATATATCTTGTGGAAAGGACGAAACACCGTGAATCTAGCTCACTAG
CTCGTTTTAGAGCTAGAAATAGCAAGTTAAAATAAGGCTAGTCCGTTATCAACTTGAAAAAG
TGGCACCGAGTCGGTGCTTTTTTGTTTTAGAGCTAGAAATAGCAAGTTAAAATAAGGCTAGT
CCGTTTTTAGCGCGTGCGCCAATTCTGCAGACAAATGGCTCTAGAGAGGGCCTATTTCCCA
TGATTCCTTCATATTTGCATATACGATACAAGGCTGTTAGAGAGATAATTGGAATTAATTTG
ACTGTAAACACAAAGATATTAGTACAAAATACGTGACGTAGAAAGTAATAATTTCTTGGGTA
GTTTGCAGTTTTAAAATTATGTTTTAAAATGGACTATCATATGCTTACCGTAACTTGAAAGT
ATTTCGATTTCTTGGCTTTATATATCTTGTGGAAAGGACGAAACACCGGTAGCTGCTATCTC
AGAGGCGTTTTAGAGCTAGAAATAGCAAGTTAAAATAAGGCTAGTCCGTTATCAACTTGAA
AAAGTGGCACCGAGTCGGTGCTTTTTTGTTTTAGAGCTAGAAATAGCAAGTTAAAATAAGG
CTAGTCCGTTTTTAGCGCGTGCGCCAATTCTGCAGACAAATGGCTCTAGAGAGGGCCTATT
TCCCATGATTCCTTCATATTTGCATATACGATACAAGGCTGTTAGAGAGATAATTGGAATTA
ATTTGACTGTAAACACAAAGATATTAGTACAAAATACGTGACGTAGAAAGTAATAATTTCTT
GGGTAGTTTGCAGTTTTAAAATTATGTTTTAAAATGGACTATCATATGCTTACCGTAACTTG
AAAGTATTTCGATTTCTTGGCTTTATATATCTTGTGGAAAGGACGAAACACCGTACTGTCTA
ATTTTTCTTCTGTTTTAGAGCTAGAAATAGCAAGTTAAAATAAGGCTAGTCCGTTATCAACT
TGAAAAAGTGGCACCGAGTCGGTGCTTTTTTGTTTTAGAGCTAGAAATAGCAAGTTAAAATA
AGGCTAGTCCGTTTTTAGCGCGTGCGCCAATTCTGCAGACAAATGGCTCTAGAGAGGGCCT
ATTTCCCATGATTCCTTCATATTTGCATATACGATACAAGGCTGTTAGAGAGATAATTGGAA
TTAATTTGACTGTAAACACAAAGATATTAGTACAAAATACGTGACGTAGAAAGTAATAATTT
CTTGGGTAGTTTGCAGTTTTAAAATTATGTTTTAAAATGGACTATCATATGCTTACCGTAAC
TTGAAAGTATTTCGATTTCTTGGCTTTATATATCTTGTGGAAAGGACGAAACACCGCTCCGT
CTAAGGAGCTGGAAGTTTTAGAGCTAGAAATAGCAAGTTAAAATAAGGCTAGTCCGTTATC
AACTTGAAAAAGTGGCACCGAGTCGGTGCTTTTTTGTTTTAGAGCTAGAAATAGCAAGTTAA
AATAAGGCTAGTCCGTTTTTAGCGCGTGCGCCAATTCTGCAGACAAATGGCTCTAGAGAGG
GCCTATTTCCCATGATTCCTTCATATTTGCATATACGATACAAGGCTGTTAGAGAGATAATT
GGAATTAATTTGACTGTAAACACAAAGATATTAGTACAAAATACGTGACGTAGAAAGTAATA
ATTTCTTGGGTAGTTTGCAGTTTTAAAATTATGTTTTAAAATGGACTATCATATGCTTACCGT
AACTTGAAAGTATTTCGATTTCTTGGCTTTATATATCTTGTGGAAAGGACGAAACACCGTCC
CGCCTAGAAGCGCAGCCGTTTTAGAGCTAGAAATAGCAAGTTAAAATAAGGCTAGTCCGTT
ATCAACTTGAAAAAGTGGCACCGAGTCGGTGCTTTTTTGTTTTAGAGCTAGAAATAGCAAGT
TAAAATAAGGCTAGTCCGTTTTTAGCGCGTGCGCCAATTCTGCAGACAAATGGCTCTAGAC
AGAACTAGTAATAGTAATCAATTACGGGGTCATTAGTTCATAGCCCATATATGGAGTTCCGA
ATAGTAATCAATTACGGGGTCATTAGTTCATAGCCCATATATGGAGTTCCGAATAGTAATCA
ATTACGGGGTCATTAGTTCATAGCCCATATATGGAGTTCCG

HEK293T Negative Control（HEK293T-CBE Clone 1#）

FlowJo 10.6.2

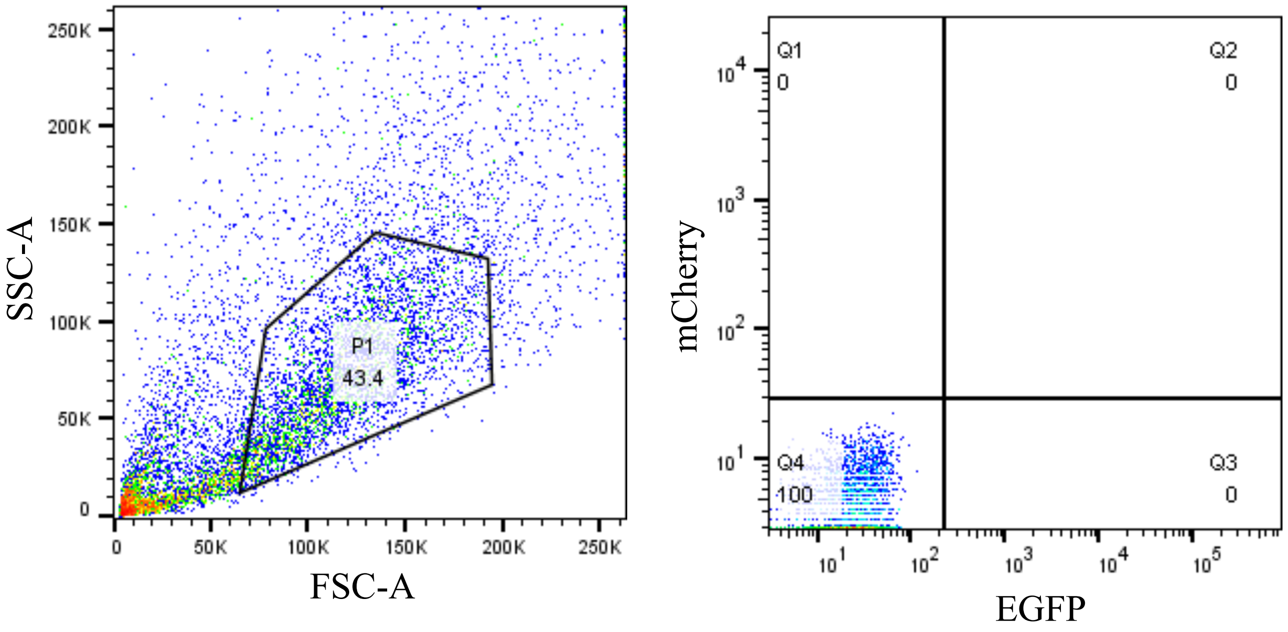

Method_1 in Figure 3 and supplementary Figure 6

FlowJo 10.6.2

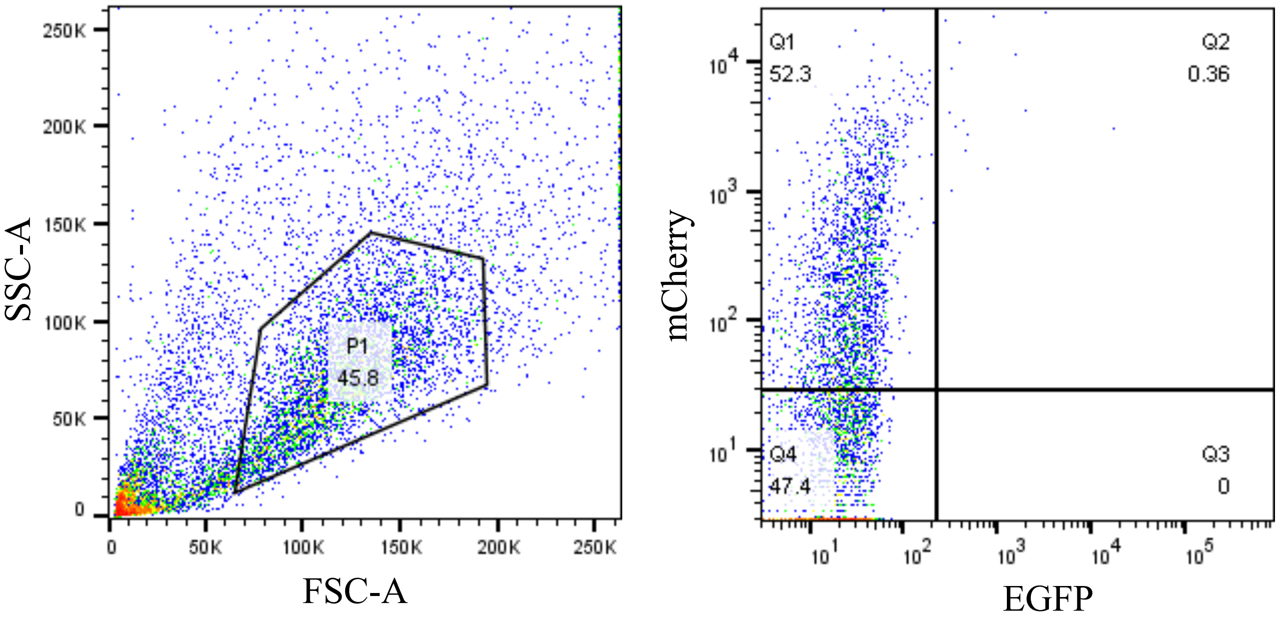

Supplementary Note. FACS gating examples for mCherry cell sorting conditions.

Method_3 in Figure 3 and supplementary Figure 6

FlowJo 10.6.2

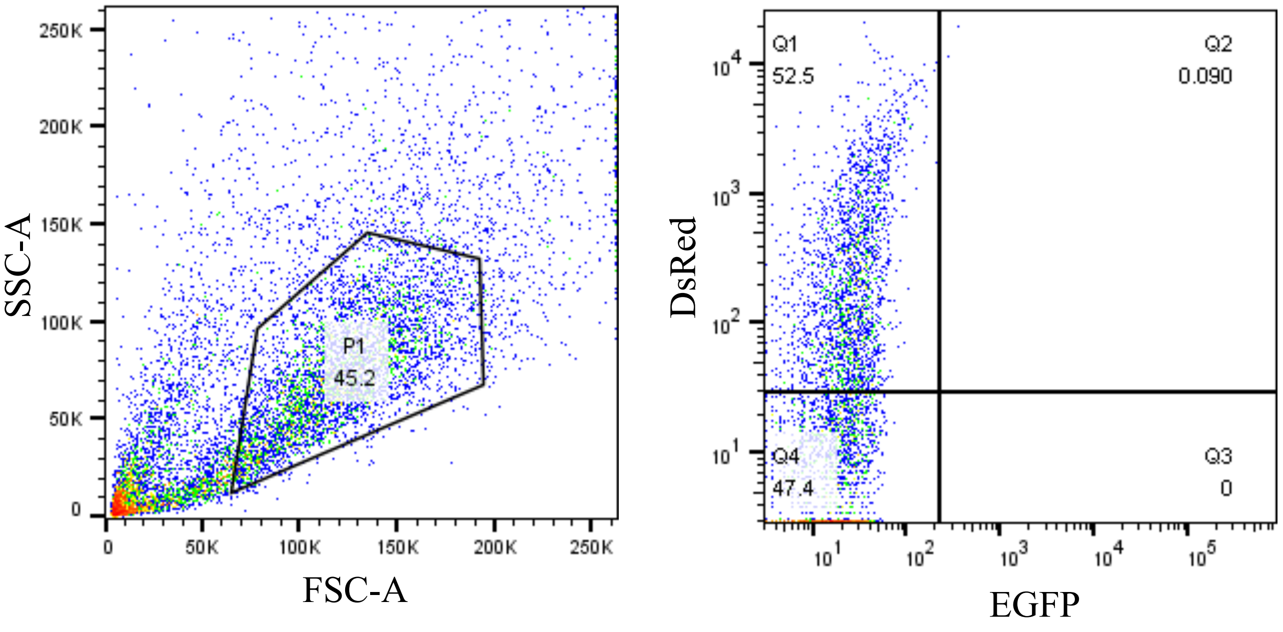

Supplementary Note. FACS gating examples for DsRed cell sorting conditions.

HEK293T Negative Control（HEK293T-CBE Clone 1#）

FlowJo 10.6.2

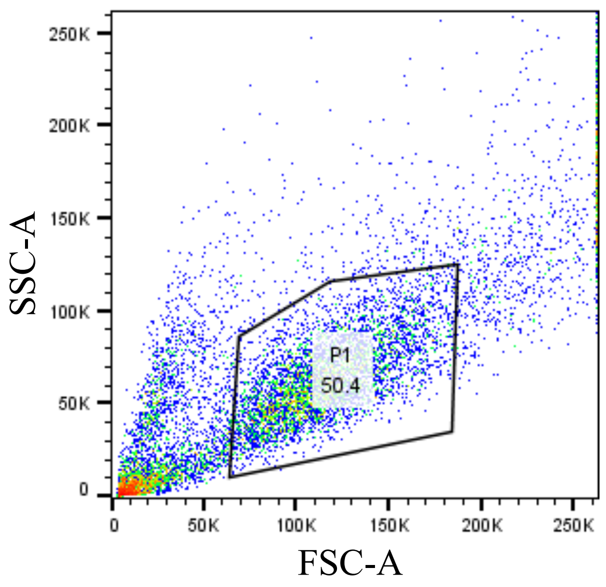

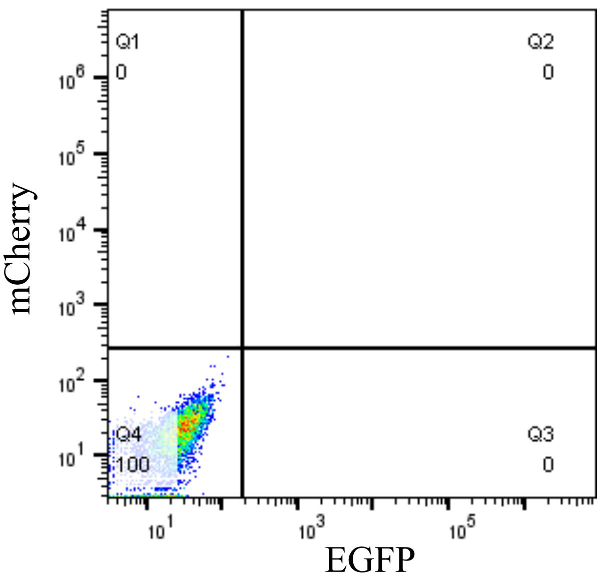

Method_2 in Figure 3 and supplementary Figure 6

FlowJo 10.6.2

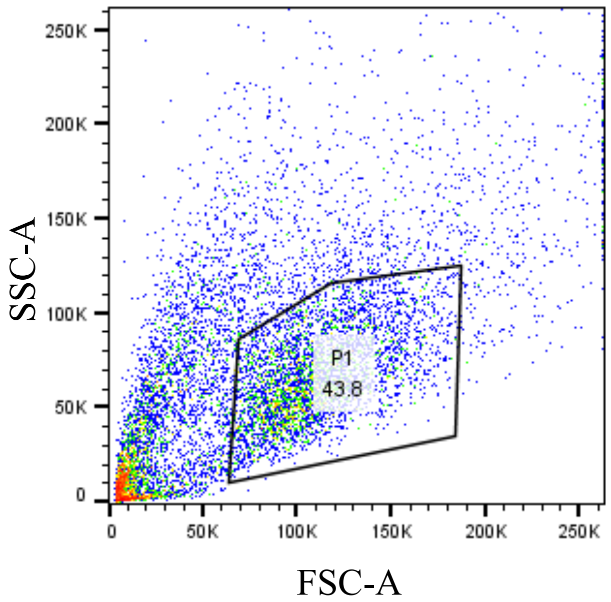

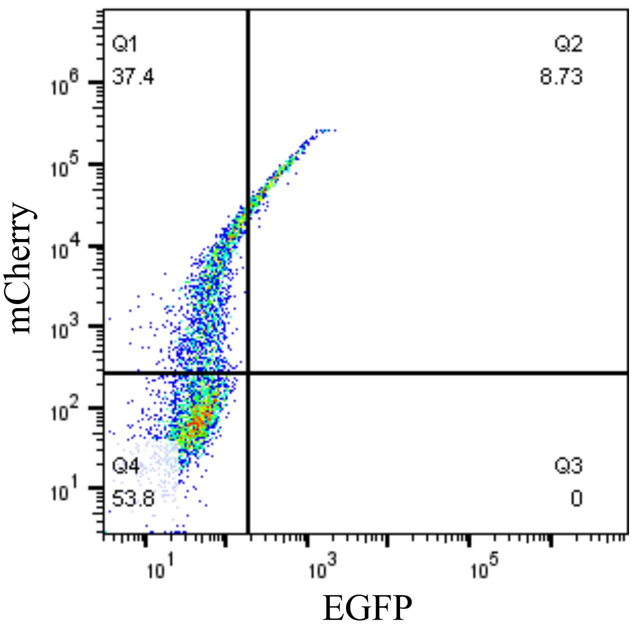

Single cell sorting in Figure 4

FlowJo 10.6.2

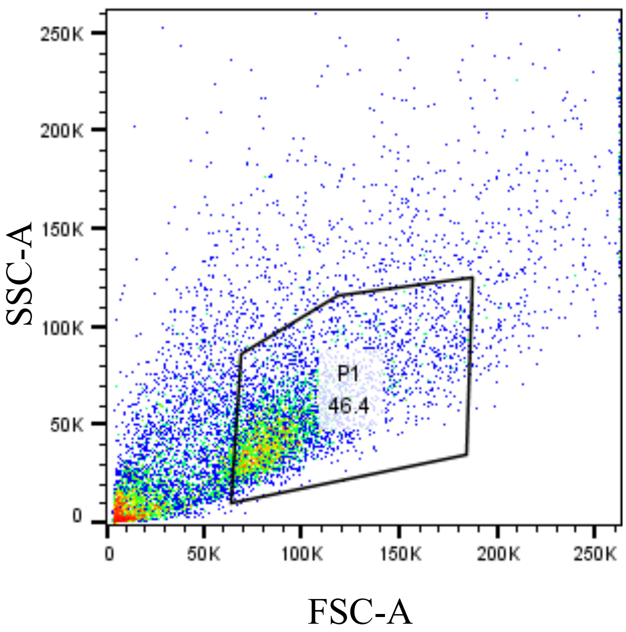

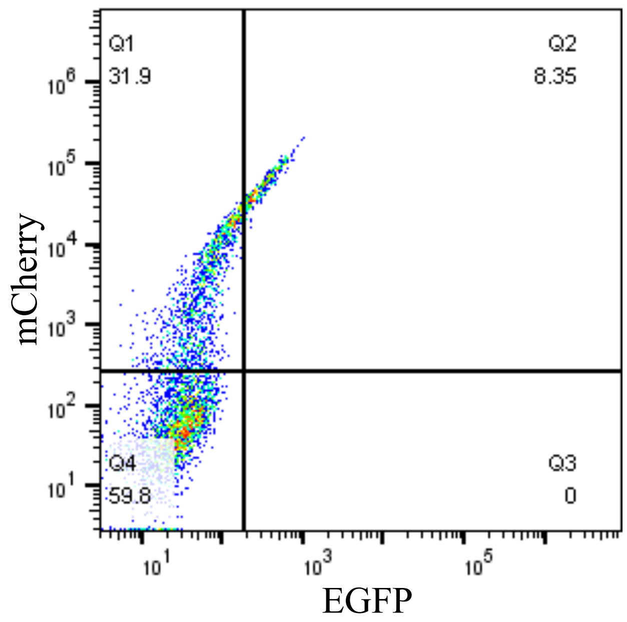

Supplementary Note. FACS gating examples for mCherry and EGFP double positive cell sorting conditions.

12h after transfection

CytExpert 2.4.0.28

Blank

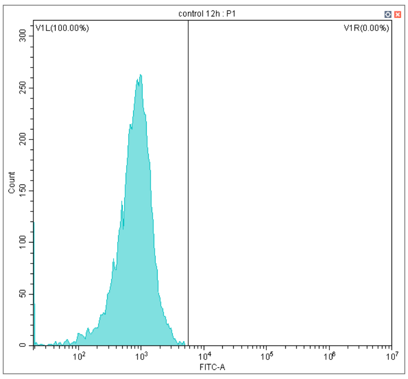

EGFP

Control

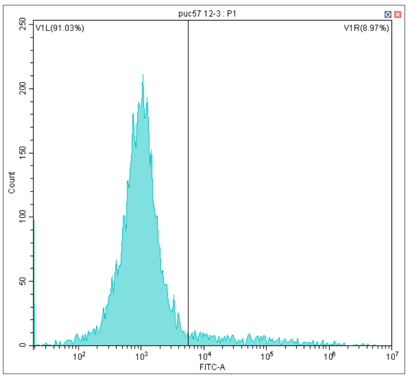

EGFP

gBlock-PC

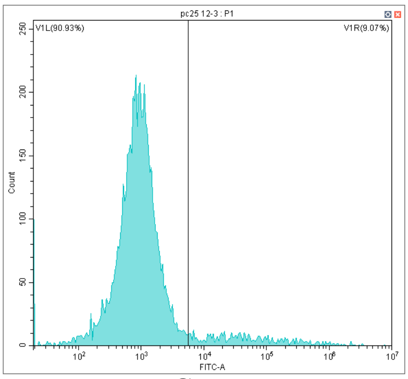

EGFP

gBlock-YC1

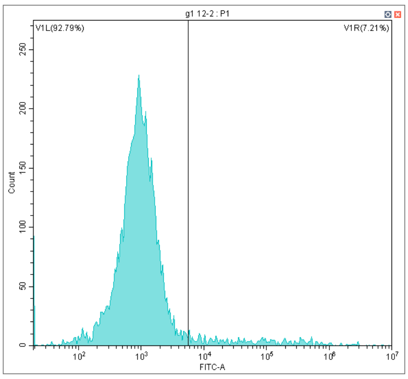

EGFP

24h after transfection

CytExpert 2.4.0.28

Blank

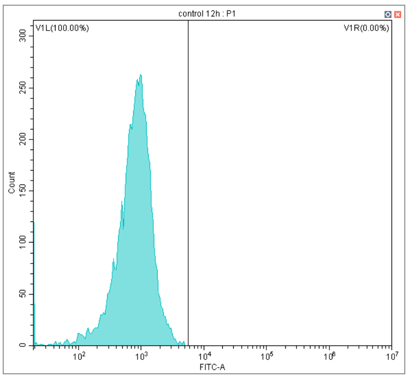

EGFP

(Same control sample with 12h)

Control

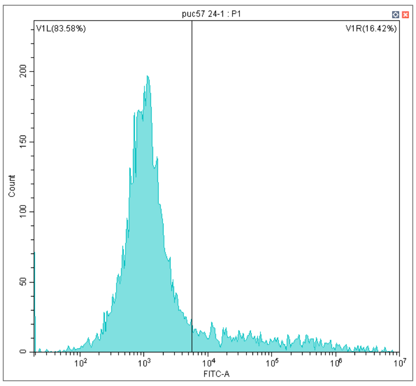

EGFP

gBlock-PC

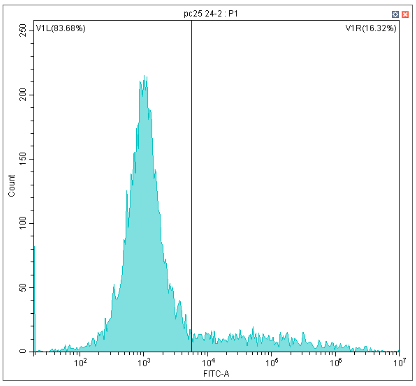

EGFP

gBlock-YC1

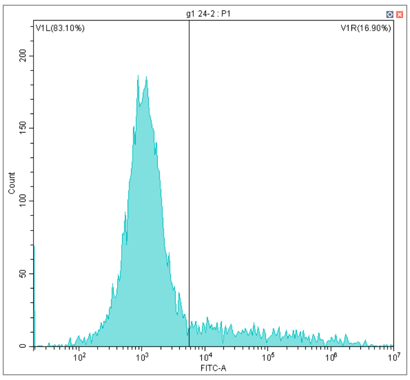

EGFP

Supplementary Note. FACS gating examples for EGFP cell sorting conditions in Supplementary Figure 3b.

48h after transfection

CytExpert 2.4.0.28

Blank

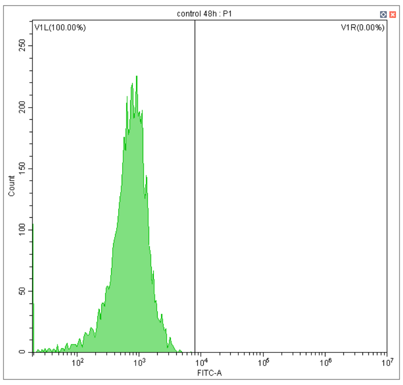

EGFP

Control

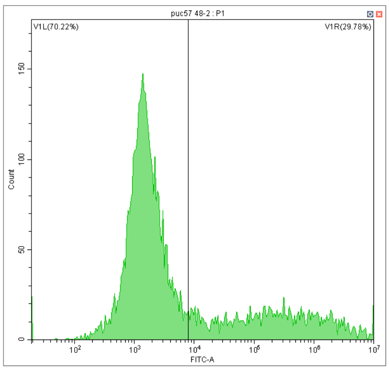

EGFP

gBlock-PC

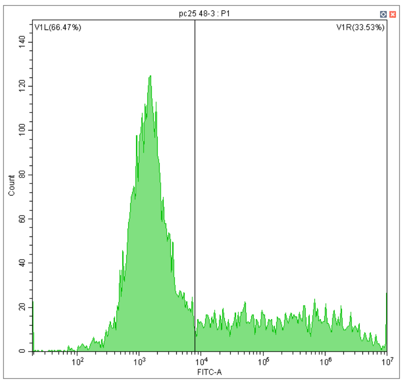

EGFP

gBlock-YC1

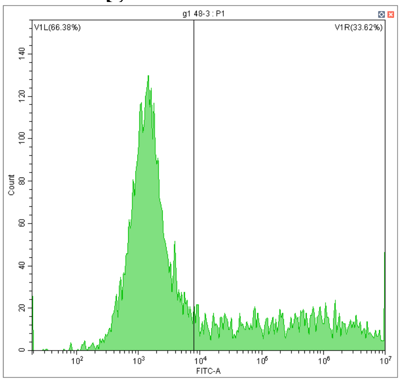

EGFP

72h after transfection

CytExpert 2.4.0.28

Blank

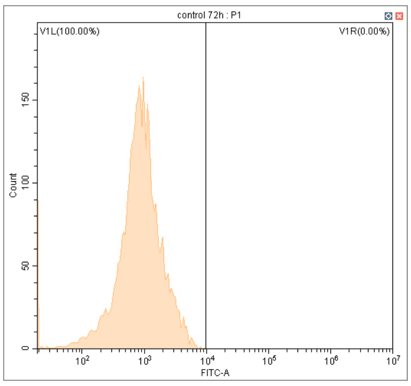

EGFP

Control

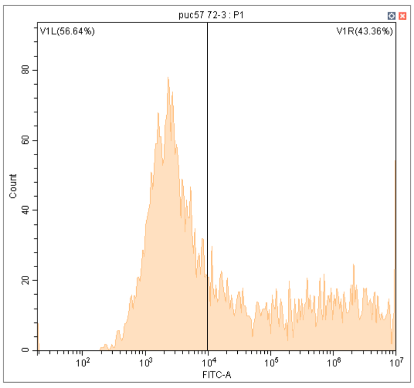

EGFP

gBlock-PC

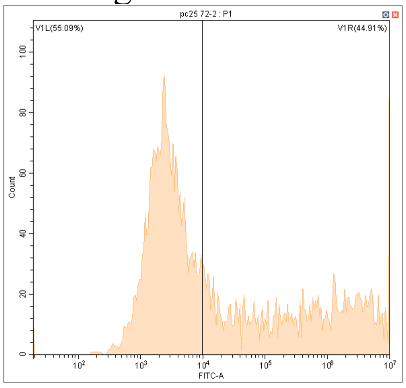

EGFP

gBlock-YC1

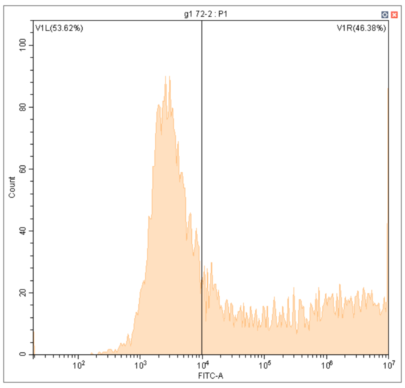

EGFP

Supplementary Note. FACS gating examples for EGFP cell sorting conditions in Supplementary Figure 3b.
