# Supplementary material for: Multiplex base editing to convert TAG into TAA codons in the human genome
Source: Nat Commun. 2022 Aug 2;13:4482. doi: 10.1038/s41467-022-31927-8 (PMC9345975; doi:10.1038/s41467-022-31927-8)
Supplement: Supplementary file 2 — Description of Additional Supplementary Files [file 41467_2022_31927_MOESM2_ESM.pdf]

**Title:** Supplementary Data 1:
**Description:** 152 sgRNAs sequences targeting genes

**Title:** Supplementary Data 2:
**Description:** Single cell RNAseq analysis for targeted C ·G to T·A editing percentage by CRISPResso2

**Title:** Supplementary Data 3:
**Description:** Whole genome sequence analysis for somatic total SNVs by mutect2 and strelka2

**Title:** Supplementary Data 4:
**Description:** Whole genome sequence analysis for Exonic SNVs in essential gene excluded targeting gene by mutect2 and strelka2

**Title:** Supplementary Data 5:
**Description:** Whole genome sequence analysis for somatic total indels by mutect2 and strelka2
